# Lysophosphatidylcholine 18:2 exacerbates Th17-dominant inflammation in obese asthma

**DOI:** 10.1186/s12944-026-02907-4

**Published:** 2026-02-24

**Authors:** Liting Cao, Huan liu, Ying Shang, Zemin Li, Yingying Ge, Tingting Hu, Abudureyimujiang Aili, Chun Chang

**Affiliations:** 1 Department of Respiratory and Critical Care Medicine, Peking University Third Hospital, 49 North Garden Road, Haidian District, Beijing, 100191 China; 2 Department of Medical Oncology and Radiation Sickness, Peking University Third Hospital, Beijing, 100191 China

**Keywords:** Lysophosphatidylcholine, Obese asthma, Th17 cells, Lipid metabolism

## Abstract

**Background:**

Obese asthma is increasingly recognized as a distinct clinical phenotype, often associated with greater disease severity and non–type 2–skewed inflammation. Obesity-associated lipid dysregulation may contribute to its pathogenesis through immunomodulatory mechanisms. This study aimed to characterize the glycerophospholipid profile of obese asthma and to investigate the functional impact of candidate lipids on immune responses.

**Methods:**

We performed targeted lipidomic analysis of serum glycerophospholipids in patients with overweight/obese asthma and those with normal-weight asthma. A house dust mite (HDM)–induced asthma model was established in diet-induced obese (DIO) mice to evaluate pulmonary pathology and T-cell immune polarization. The immunomodulatory effects of lysophosphatidylcholine (LPC) 18:2 were assessed using in vivo experiments and in vitro T helper 17 (Th17) differentiation assays. Cellular uptake of LPC by naïve CD4⁺ T cells was quantified to explore the mechanism underlying the reduced circulating abundance of LPC 18:2.

**Results:**

Lipidomic profiling revealed a differential glycerophospholipid signature in obese asthma, with LPC 18:2 exhibiting the most pronounced reduction. Obese asthmatic mice exhibited exacerbated airway inflammation consistent with a Th17-skewed immune response. Despite reduced circulating levels, administration of exogenous LPC 18:2 further aggravated airway inflammation and selectively enhanced Th17 polarization in these mice. In vitro assays confirmed that LPC 18:2 directly promotes Th17 differentiation in a dose-dependent manner. Mechanistically, naïve CD4⁺ T cells from obese mice showed increased LPC uptake, potentially explaining both the reduced circulating LPC 18:2 levels and its amplified pro-inflammatory effects in obese asthma.

**Conclusions:**

Our findings highlight LPC 18:2 as a potential metabolic regulator that may link obesity-associated lipid disturbances to pathological Th17-skewed immune responses. Modulation of LPC 18:2–related pathways may therefore represent a novel therapeutic strategy for this asthma phenotype.

**Supplementary Information:**

The online version contains supplementary material available at 10.1186/s12944-026-02907-4.

## Background

The Global Burden of Disease (GBD) Study ranks asthma as one of the most prevalent chronic respiratory diseases worldwide, affecting over 260 million individuals and contributing to approximately 40 million incident cases annually [1]. Among its recognized risk factors, obesity has emerged as a major contributor [2–4] and is strongly associated with greater asthma severity [5, 6]. Obese asthma has been estimated to account for more than half of all severe or refractory cases [7]. Clinically, obese asthma presents distinct features, including reduced lung function, heightened symptom burden, poorer quality of life, increased exacerbations, and a diminished response to corticosteroid therapy [8, 9]. Moreover, conventional biomarkers often show reduced diagnostic performance in obese individuals [10], underscoring obese asthma as a distinct phenotype driven by specific pathogenic mechanisms that warrant further investigation.

Unlike classical Th2-high asthma, obese asthma is predominantly a non–type 2 inflammatory disorder, characterized by a Th1/Th17-skewed immune response [11]. Increased Th17 cell infiltration and elevated IL-17A levels have been consistently observed in obese asthma [12, 13]. Obesity also intensifies Th17-driven inflammation in other chronic inflammatory diseases, such as multiple sclerosis and inflammatory bowel disease [14], suggesting that similar mechanisms may operate in asthma. However, the mechanisms linking obesity to Th17 skewing remain poorly understood.

Obesity represents a state of systemic metabolic dysregulation, accompanied by profound disturbances in lipid metabolism [15]. Metabolomic studies have repeatedly identified significant alterations in glycerophospholipid (GP) profiles in obese individuals, which have been implicated in lipotoxic injury associated with various metabolic diseases [16–18]. Notably, obese asthmatics have been reported to exhibit distinct lipidomic signatures, including a unique respiratory metabolomic profile absent in both lean asthmatics and obese non-asthmatic controls [19]. Despite these observations, the immunological consequences of such lipid remodeling—particularly its impact on T cell–mediated immunity—remain poorly defined.

As fundamental components of biological membranes, GPs have recently emerged as potent immunoregulatory mediators that influence T cell fate and function [20]. This regulation occurs in part through enzymatic hydrolysis of GPs into lysophospholipids [21–23]. These bioactive lipids modulate lipid raft composition, influence T cell receptor (TCR) signaling [24], and regulate key metabolic pathways that guide lineage differentiation [25], thereby potentially contributing to asthma pathogenesis [20, 23]. Given their established immunoregulatory roles, dysregulated GP metabolism may represent a key mechanistic link between obesity-associated lipid disturbances and immune dysfunction in asthma.

In this study, lipidomic profiling revealed a distinct glycerophospholipid signature in obese asthma, marked by a reduction in LPC 18:2 levels compared with non-obese asthma. Functional analyses demonstrated that LPC 18:2 exacerbates airway inflammation and selectively enhances Th17 responses specifically under obese conditions. We further found that increased LPC 18:2 uptake by naïve CD4⁺ T cells in obesity may contribute to enhanced Th17 polarization, offering a plausible explanation for its reduced circulating levels. Together, these findings support a role for LPC 18:2 in promoting Th17-biased inflammation, provide new mechanistic insights into obese asthma pathogenesis, and suggest potential targets for therapeutic intervention.

## Methods

### Study subjects

A total of 50 consecutive patients with asthma and 49 healthy controls were recruited at the Department of Respiratory Medicine, Peking University Third Hospital. Asthma was diagnosed according to the Global Initiative for Asthma (GINA) strategy [26]. Exclusion criteria included a recent asthma exacerbation (within 4 weeks), other chronic respiratory diseases, chronic comorbidities (severe cardiovascular, hepatic, renal, hematologic, autoimmune diseases, or malignancy), metabolic disorders (except obesity), pregnancy, or inability to provide informed consent. Patients who had received systemic corticosteroids or antibiotics within 2 weeks prior to enrollment were also excluded. Healthy controls were screened to exclude any history of respiratory, allergic, metabolic disorders (other than obesity), or immune disorders. The study protocol was approved by the Institutional Review Board of Peking University Third Hospital (Ethics Approval No. M2020023) and conducted in accordance with the Declaration of Helsinki. Written informed consent was obtained from all participants prior to enrollment.

### Glycerophospholipid profiling

Targeted lipidomic profiling was performed using Ultra-Performance Liquid Chromatography–Tandem Mass Spectrometry (UPLC–MS/MS). Serum samples (100 µL) were extracted using a modified methyl tert-butyl ether (MTBE) method. Briefly, samples were combined with 400 µL ice-cold 80% methanol containing a mixture of internal standards (including phosphatidylcholine PC 34:0 at a final concentration of 0.2 ng/mL) and 1 mL MTBE. Phase separation was induced by adding 250 µL ddH₂O and incubating for 10 min at room temperature. Following centrifugation (12,000 × g, 10 min, 4 °C), the upper organic phase was collected and evaporated to dryness under a gentle nitrogen stream. Lipid residues were reconstituted in 150 µL HPLC-grade methanol, vortexed for 10 min, and centrifuged; the resulting supernatant was injected into the UPLC–MS/MS system (1 µL for positive mode and 5 µL for negative mode).

Chromatographic separation was achieved on an ACQUITY UPLC BEH C18 column (1.7 µm, 100 × 2.1 mm; Waters, Milford, MA, USA) using an ACQUITY UPLC system (Waters). Detection was performed on a 5500 QTRAP mass spectrometer (AB Sciex, Framingham, MA, USA) equipped with a Turbo Ion Spray electrospray ionization source. Data were acquired in multiple reaction monitoring (MRM) mode with the following source parameters: curtain gas (CUR) 40 psi; ion source gas 1, 30 psi; ion source gas 2, 30 psi; ion spray voltage + 5500 V (positive mode)/−4500 V (negative mode); collision gas, medium; source temperature, 350 °C. Full details of MRM transitions, internal standards, calibration curves, quality-control procedures, and processed lipidomics data are provided in the Supplementary Materials (Additional file 1 and Additional file 2).

Raw data were processed using Analyst 1.6 and quantified with MultiQuant 3.0.2. Lipid species were identified by retention time alignment with internal standards and relative quantification was performed by peak area ratios. Subsequent statistical analysis was performed using GraphPad Prism 10.0 and MetaboAnalyst 6.0 at http://www.metaboanalyst.ca. Peak areas were first normalized to their corresponding internal standards and then scaled by the total lipid signal per sample (total-sum normalization). Features with missing values in > 50% of samples were removed, and remaining missing values were imputed in MetaboAnalyst using k-nearest neighbors (k = 5) prior to downstream analysis.

### Mice and experimental model

6-week-old male C57BL/6J wild-type mice were obtained from the Department of Laboratory Animal Science, Peking University Health Science Center. Animals were housed under specific pathogen-free (SPF) conditions with free access to food and water, at 21–24 °C, 40–70% relative humidity and a 12-hour light/dark cycle (lights on 07:00–19:00). All animal procedures were approved by the Ethics Committee of Peking University Third Hospital (Ethics Approval No. LA2020391).

To establish the diet-induced obesity model, mice were acclimated for 1 week and then randomly assigned to be fed either a normal chow diet (NCD; XTCON50J) or a high-fat diet (HFD; XTHF60) for 12 weeks. Mice were monitored weekly for body weight and general health.

Before intranasal instillation and intraperitoneal injection procedures, mice were anaesthetized using isoflurane (RWD Life Science, R510-22-10) delivered via a vaporizer (approximately 2% isoflurane in oxygen at a flow rate of 2 L/min for 30–60 s) until loss of the righting reflex. Anaesthesia was induced but not maintained because the procedures were brief. During anaesthesia, respiration, mucous membrane color, and general condition were closely monitored. After the procedures, mice were placed on a warm pad and observed until full recovery of mobility and normal behavior.

To induce experimental asthma, mice were sensitized by intranasal (i.n.) administration of 10 µg HDM extract (Stallergenes Greer, XPB82D3A25) on day 0, followed by daily intranasal challenges with 10 µg HDM from day 7 to day 13. Control mice received PBS on the same schedule.

For LPC 18:2 administration, treatment groups received intraperitoneal (i.p.) injections of LPC 18:2 (MedChemExpress, HY-N9410) at 10 mg/kg every other day from day 1 to day 13 (seven doses in total), while control groups received vehicle (normal saline containing the same final DMSO concentration as the LPC 18:2 formulation) on the same schedule. LPC 18:2 was prepared as a 50 mM stock solution in DMSO and diluted with normal saline to a final concentration of 2 mg/mL before injection.

Mice were euthanized 24 h after the final HDM challenge. For euthanasia, mice were deeply anaesthetized with isoflurane (induction at approximately 5% in oxygen) until loss of the pedal reflex was confirmed. Trained personnel then performed cervical dislocation to ensure rapid and humane euthanasia. The left lung was fixed in 10% neutral-buffered formalin, embedded in paraffin, and sectioned at 5 µm. Lung sections were stained with hematoxylin and eosin (H&E) or periodic acid–Schiff (PAS) reagents. Airway inflammation and goblet cell hyperplasia were scored independently by two blinded observers according to established criteria.

H&E scoring criteria: 0 (No inflammatory cells), 1 (Occasional inflammatory cells), 2 (1–3 layers of inflammatory cells), 3 (4–5 layers of inflammatory cells), 4 (> 5 layers of inflammatory cells).

PAS scoring criteria based on the percentage of goblet cells in the epithelium: 0 (No goblet cells), 1 (< 25% goblet cells), 2 (25–50% goblet cells), 3 (51–75% goblet cells), 4 (> 75% goblet cells or mucus-filled lumen) [27]. The mean score per airway was calculated by dividing the total score by the number of airways examined.

### Naïve CD4⁺ T cell isolation and differentiation

Naïve CD4⁺ T cells were isolated from splenic single-cell suspensions of C57BL/6J mice using the Naïve CD4⁺ T Cell Isolation Kit (Mouse; Miltenyi, 130-104-453), according to the manufacturer’s instructions. Magnetic separation was performed manually with MACS^®^ columns. Purified cells were cultured in KBM581 lymphocyte medium (Corning, 88–581-CM) supplemented with 10% fetal bovine serum (Sigma-Aldrich, F8318) and 1% penicillin–streptomycin (Gibco, 15140-122). Cells were activated with plate-bound anti-CD3 (10 µg/mL; BioLegend, 100340; plates coated overnight at 4 °C) and soluble anti-CD28 (10 µg/mL; BioLegend, 102116). Naïve CD4⁺ T cells were polarized under lineage-specific conditions: Th1 (recombinant IL-2 10 ng/mL, murine IL-12 20 ng/mL, anti-IL-4 10 µg/mL), Th2 (recombinant IL-2 10 ng/mL, murine IL-4 10 ng/mL, anti-IFN-**γ** 10 µg/mL), Th17 (recombinant TGF-β1 3 ng/mL, murine IL-6 20 ng/mL, murine IL-1β 10 ng/mL, murine IL-23 10 ng/mL, anti-IFN-**γ** 10 µg/mL, anti-IL-4 10 µg/mL), and Treg (recombinant TGF-β1 5 ng/mL, recombinant IL-2 10 ng/mL, anti-IFN-**γ** 10 µg/mL, anti-IL-4 10 µg/mL). LPC 18:2 (0, 10, 20, 50 µM; dissolved in DMSO) was added at culture initiation; final DMSO concentration was maintained at 0.1% (v/v) across all conditions. Cells were incubated at 37 °C with 5% CO₂ and harvested after 72 h for intracellular cytokine staining and flow cytometric analysis. Typical seeding density was 1 × 10⁵ cells/well in a 96-well plate. Experiments were performed with at least three independent biological replicates.

For pharmacological inhibition of the autotaxin (ATX/ENPP2)–lysophosphatidic acid (LPA) axis, naïve CD4⁺ T cells were cultured under Th17-polarizing conditions as described above, with or without LPC 18:2 (50 µM), in the presence or absence of the autotaxin inhibitor PF-8380 (0.1 or 1 µM; MedChemExpress, HY-13344) and/or LPA receptor antagonists. LPA₁–₃ signaling was blocked using a combination of Ki16425 (LPA₁/₃ antagonist; MedChemExpress, HY-13285) and H2L5186303 (LPA₂ antagonist; MedChemExpress, HY-107616) at 1 or 5 µM each. All pharmacological agents were dissolved in DMSO, and the final DMSO concentration was kept constant across all treatment and control groups. Th17 differentiation was assessed after 72 h by flow cytometric analysis.

### Flow cytometry

Fluorochrome-conjugated antibodies used in this study (all from BioLegend) included: Alexa Fluor 700 anti-mouse CD3 (100216), PerCP/Cyanine5.5 anti-mouse CD4 (100434), PE/Dazzle™ 594 anti-mouse CD8a (100761), PE/Cyanine7 anti-mouse CD25 (102015), PE/Dazzle™ 594 anti-mouse/human CD44 (103055), PE/Cyanine7 anti-mouse CD62L (104417), PE anti-mouse IFN-**γ** (163503), APC anti-mouse IL-4 (504105), Brilliant Violet 605™ anti-mouse IL-17A (506927), and Brilliant Violet 421™ anti-mouse Foxp3 (126419). Isotype-matched control antibodies included Brilliant Violet 605 Rat IgG1, κ (400433) and PerCP/Cyanine5.5 Rat IgG2b, κ (400631), which were included in selected experiments as isotype controls for the IL-17A and CD4 channels, respectively, to assess non-specific binding.

Cells were resuspended in PBS and stained with the Zombie Aqua Fixable Viability Kit (BioLegend, 423101) to exclude dead cells. For intracellular cytokine staining, cells were stimulated with Cell Activation Cocktail (BioLegend, 423302) for 1 h, followed by the addition of brefeldin A (BFA; BioLegend, 420601) and further incubation for 4 h (total 5 h). Surface staining was performed first, followed by fixation, permeabilization and intracellular cytokine staining. For transcription factor staining, cells were fixed and permeabilized with Foxp3/Transcription Factor Staining Buffer Set (Invitrogen, 00–5523-00) and then incubated with the appropriate antibodies prior to analysis. Fluorescence-minus-one (FMO) controls were used for intracellular markers to define positive gates. Each FMO sample contained the full antibody panel except for the marker of interest, and gates were set based on the corresponding FMO distributions to distinguish positive events from background. In selected experiments, isotype-matched control antibodies were included to assess non-specific binding and confirm staining specificity (Supplementary Fig. S1).

T helper cell subsets were defined as follows in animal experiments: Th1 cells as CD3⁺CD4⁺CD8⁻IFN-γ⁺, Th2 as CD3⁺CD4⁺CD8⁻IL-4⁺, Th17 as CD3⁺CD4⁺CD8⁻IL-17A⁺, and Treg as CD3⁺CD4⁺CD25⁺Foxp3⁺. For naïve CD4⁺ T cell differentiation assays, high-purity naïve CD4⁺ T cells were pre-enriched, and the frequencies of IFN-γ⁺, IL-4⁺, IL-17A⁺, and Foxp3⁺ cells among viable cells were used to define Th1, Th2, Th17, and Treg subsets, respectively. Flow cytometry data were acquired using a Sony ID7000 spectral analyzer and a Beckman CytoFLEX flow cytometer depending on the experiment.

### Transcriptome sequencing and analysis

Transcriptome sequencing was performed by Beijing Novogene Bioinformatics Technology Co., Ltd. RNA integrity and purity were assessed using an Agilent 2100 Bioanalyzer (Agilent Technologies, CA, USA). Poly(A)⁺ mRNA was enriched from total RNA using Oligo(dT) magnetic beads, followed by library construction according to the manufacturer’s standard protocol. The libraries were sequenced on the Illumina HiSeq platform to generate paired-end reads. Raw reads were subjected to quality control and adapter trimming using fastp to obtain high-quality clean data. Clean reads were aligned to the *Mus musculus* reference genome (GRCm38/mm10) using a standard RNA-seq analysis pipeline. Gene-level read counts were quantified with featureCounts (v1.5.0-p3). Differential gene expression analysis was conducted using the DESeq2 package (v1.20.0) in R, with significance defined as adjusted *P* (Padj) < 0.05 and |fold change (FC)| > 1.5. Functional enrichment analyses of differentially expressed genes were performed using the clusterProfiler package (v3.8.1). Kyoto Encyclopedia of Genes and Genomes (KEGG) pathway analysis was applied to identify significantly enriched biological pathways.

### LPC uptake assay

Naïve CD4⁺ T cells were isolated from the spleens of obese and normal-weight male mice as previously described. Cells were activated in vitro with plate-bound anti-CD3 and soluble anti-CD28 antibodies for 72 h, with unstimulated cells serving as controls. Following activation, cells were washed with PBS and preincubated for 1 h in KBM581 lymphocyte medium (Corning, 88–581-CM) supplemented with 0.5% fatty acid-free BSA (Sigma-Aldrich, A1595) to minimize interference from endogenous lipids. Cells were then incubated with TopFluor–LPC (Avanti Polar Lipids, 810284) at 1, 2, 5, or 10 µM for 30 min at 37 °C. After incubation, cells were washed three times with ice-cold PBS to terminate uptake and then analyzed by flow cytometry. LPC uptake was quantified as mean fluorescence intensity (MFI) in viable singlet CD4⁺ T cells.

For confocal imaging, naïve CD4⁺ T cells were incubated with 1 µM TopFluor–LPC under identical conditions, washed three times with ice-cold PBS, and fixed with 4% paraformaldehyde for 10 min. Nuclei were counterstained with Hoechst (Solarbio, C0030), and images were acquired using a Zeiss LSM confocal microscope under identical laser intensity, gain, and exposure settings across all groups. Quantitative image analysis was performed using Zeiss ZEN Blue and ImageJ (Fiji) software. Regions of interest (ROIs) were manually delineated around individual cells, and the MFI of TopFluor–LPC was determined after background subtraction. For each biological replicate, at least 50 cells were analyzed, and the average MFI per mouse was used for statistical analysis. Detailed descriptions of other experimental methods are available in the supplementary information (Additional file 3).

### Statistical analysis

Statistical analyses and data visualization were performed using SPSS version 27.0 and GraphPad Prism version 10.0. Data distribution was assessed using the Kolmogorov–Smirnov test. Continuous variables following an approximately normal distribution were presented as mean ± standard deviation (SD), whereas non-normally distributed variables were reported as median (interquartile range, IQR). For normally distributed data, group comparisons were conducted using an unpaired two-tailed *t*-test, one-way ANOVA, or repeated-measures ANOVA, as appropriate. For non-normally distributed data, comparisons were performed using the Mann–Whitney *U* test or Kruskal–Wallis test. Chi-square or Fisher’s exact tests were used for categorical variables. Correlations between continuous variables were analyzed using Pearson’s correlation coefficient for normally distributed data and Spearman’s rank correlation coefficient for non-normally distributed or ordinal data. For subgroup analyses, asthma control was defined as controlled (Asthma Control Test (ACT) score ≥ 20) versus uncontrolled (ACT score < 20); FeNO was dichotomized at 50 ppb (low vs. high), and blood eosinophils were categorized as low (< 150 cells/µL) versus high (≥ 150 cells/µL). FEV₁% predicted was categorized as ≥ 80% versus < 80%, and FEV₁/FVC as ≥ 70% versus < 70%. For lipidomic volcano plots, predefined thresholds for differential abundance were set at |fold change| > 1.2 with nominal *P* < 0.05 for the exploratory human dataset and |fold change| > 2.0 with false discovery rate (FDR)–adjusted *P* < 0.05 for the confirmatory murine dataset. All statistical tests were two-sided, and differences were considered statistically significant at *P* < 0.05.

## Results

### Comparative analysis of clinical profiles and inflammatory cytokine patterns across study groups

A total of 50 patients with asthma (24 males, 26 females; mean age 47.0 ± 16.1 years) and 49 healthy controls (23 males, 26 females; mean age 48.3 ± 16.3 years) were enrolled. Participants were stratified by body mass index (BMI) into normal-weight (18.5 ≤ BMI < 25 kg/m²) and overweight/obese (BMI ≥ 25 kg/m²) subgroups for comparative analyses. As detailed in Table 1, age and sex distributions were comparable across groups (all *P* > 0.05). Compared with healthy controls in the same BMI category, normal-weight patients with asthma exhibited significantly reduced FEV₁/FVC ratios. In contrast, no significant differences were observed between the two asthma groups with respect to age at asthma onset, disease control status, blood eosinophil counts (BEC), total IgE, fractional exhaled nitric oxide (FeNO), or other spirometric indices. Cytokine profiling (Table 2) revealed significantly elevated IL-6 levels in overweight/obese patients with asthma compared with their normal-weight counterparts (*P* = 0.032), suggesting obesity-associated systemic inflammation, whereas the other measured cytokines (IL-4, IL-5, IL-13, IL-10, IL-17A, and IL-17F) did not differ significantly.

**Table 1:** Comparison of sociodemographic, clinical, and pulmonary function parameters across study groups

Characteristics	Normal Weight		Overweight/Obese		Overall *P*-value
	Control (*n* = 27)	Asthma (*n* = 34)	Control (*n* = 22)	Asthma (*n* = 16)	
Sociodemographic characteristics					
Age (year)	45.7 ± 17.6	48.5 ± 14.8	51.4 ± 14.4	43.9 ± 18.8	0.483
Sex (Female/male)	16/11	18/16	10/12	8/8	0.807
BMI (kg/m^2^)	22.60 (2.08)	22.67 (2.89)	27.76 (4.19) ^*§*^	27.66(4.81) ^*‡*^	<0.001
Clinical characteristics					
Age at asthma onset (year)	N/A	40.94 ± 14.56	N/A	41.25 ± 17.78	0.948
ACT score	N/A	16.47 ± 4.51	N/A	18.75 ± 4.09	0.093
Blood eosinophils counts (cells/µL)	110.00 (200.00)	315.00 (395.00)	130.00 (137.50)	260.00 (437.50)	0.010
Total IgE (IU/mL)	N/A	121.00 (241.40)	N/A	201.00 (288.00)	0.631
FeNO (ppb)	N/A	42.00 (66.00)	N/A	41.00 (83.00)	0.719
Pulmonary function test					
TLC% predicted	99.84 ± 14.66	106.68 ± 11.74	99.20 ± 11.58	106.44 ± 14.84	0.178
FVC% predicted	90.45 ± 17.12	91.96 ± 17.18	91.58 ± 15.42	92.99 ± 15.07	0.981
FEV_1_% predicted	93.00 (21.65)	80.80 (30.98)	93.00 (13.10)	88.45 (15.60)	0.130
FEV_1_/FVC ratio	82.42 ± 7.16	71.09 ± 12.00 ^*§*^	78.47 ± 4.49	75.43 ± 10.85	<0.001
RV% predicted	118.00 (46.15)	130.90 (52.50)	121.00 (47.60)	145.55 (56.55)	0.295
RV/TLC	43.18 (16.37)	39.72 (19.68)	40.24 (16.97)	39.68 (23.87)	0.888

Data are presented as mean ± SD or median (IQR). Overall group differences were assessed using one-way ANOVA with Tukey’s post hoc test or the Kruskal–Wallis test with Bonferroni-adjusted post hoc pairwise comparisons, as appropriate based on data distribution. Comparisons between two groups were conducted using an unpaired two-tailed *t*-test or the Mann–Whitney *U* test

In pairwise comparisons, ^*§*^ indicates *P* < 0.05 versus normal-weight controls; ^*‡*^ indicates *P* < 0.05 versus normal-weight asthma

**Table 2:** Comparison of serum cytokine levels between normal-weight and overweight/obese patients with asthma

Cytokines	Normal-weight asthma (pg/mL) (*n* = 26)	Overweight/Obese asthma (pg/mL) (*n* = 13)	*P*-value
IL-4	2.94 (6.43)	2.60 (4.47)	0.872
IL-5	4.08 (10.15)	4.88 (11.03)	0.622
IL-13	9.56 (17.17)	4.95 (14.70)	0.713
IL-2	1.96 3.38)	0.76 (1.36)	0.099
IL-6	7.41 (15.72)	12.22 (28.14)	0.032*
IL-9	6.96 (13.14)	4.14 (5.42)	0.219
IL-10	1.90 (1.46)	1.67 (2.62)	0.941
IFN-γ	5.95 (15.53)	5.54 (28.92)	0.803
TNF-α	12.34 (39.94)	18.58 (65.60)	0.758
IL-17A	1.37 ± 0.97	1.90 ± 2.27	0.434
IL-17F	1.61 (3.92)	1.30 (4.17)	0.384
IL-22	2.88 (2.26)	2.38 (1.62)	0.988

Data are presented as mean ± SD or median (IQR). Statistical differences were assessed using the Mann–Whitney *U* test, with IL-17A analyzed using an unpaired two-tailed *t*-test

*P < 0.05, **P < 0.01, ***P < 0.001

### LPC 18:2 downregulation is a conserved metabolic hallmark of obese asthma

**Fig. 1:**
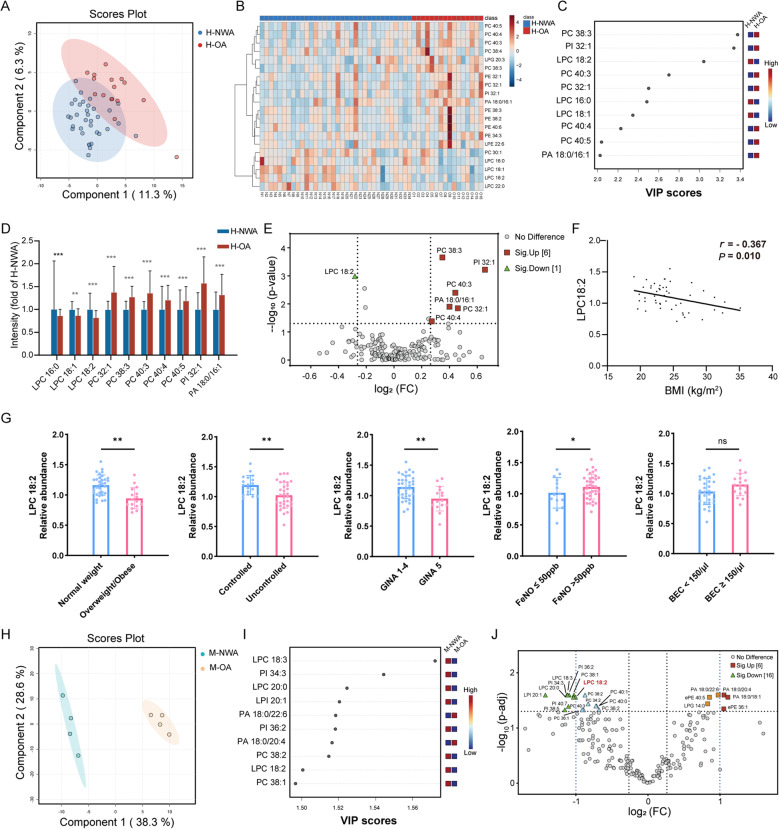
Identification and validation of LPC 18:2 reduction in obese asthma. **A–G** Discovery lipidomic profiling in human asthma patients. **A** PLS-DA score plot of serum glycerophospholipids from overweight/obese (H-OA, n = 16) and normal-weight (H-NWA, n = 34) asthmatic patients. **B** Heatmap showing the top 20 differentially abundant glycerophospholipids (Z-score scaled). **C** VIP scores of the top 10 lipids contributing to group separation in (A). **D** Relative abundances of the top 10 VIP-ranked lipid species (mean ± SD; unpaired two-tailed *t*-test). **E** Exploratory volcano plot from the human screen (nominal *P* < 0.05 and |fold change| > 1.2). LPC 18:2 showed the most pronounced decrease among candidate lipids. **F** Correlation between serum LPC 18:2 levels and BMI across the patient cohort, assessed by Pearson’s correlation coefficient. **G** Serum LPC 18:2 levels in clinical asthma subgroups: normal-weight vs. overweight/obese asthma, controlled (ACT score ≥ 20) vs. uncontrolled asthma (ACT score < 20), GINA Steps 1–4 vs. Step 5, FeNO-high vs. FeNO-low (50 ppb cutoff), and eosinophil-high vs. eosinophil-low (150 cells/µL cutoff) (mean ± SD; unpaired two-tailed *t*-test). **H–J** Independent validation in a murine model of obese asthma. **H** PLS-DA score plot of serum glycerophospholipids from obese (M-OA) and normal-weight (M-NWA) asthmatic mice (n = 4 per group). **I** VIP scores corresponding to (H). **J** Volcano plot from the confirmatory lipidomic analysis (FDR–adjusted *P* < 0.05). Vertical dashed lines denote fold change cutoffs at fold change = 2.0 (blue) and fold change = 1.2 (black). The LPC 18:2 reduction observed in humans was independently reproduced in mice, meeting both FDR significance and the stringent |fold change| > 2.0 threshold. *P < 0.05, **P < 0.01, ***P < 0.001, ns, not significant

Given the well-established association between obesity and perturbations in lipid metabolism, we performed targeted lipidomic profiling to characterize glycerophospholipid alterations specific to obese asthma. In a human discovery cohort, partial least squares discriminant analysis (PLS-DA) showed segregation between obese and non-obese asthmatics based on their glycerophospholipid profiles (Fig. 1A). Heatmap visualization (Fig. 1B) and variable importance in projection (VIP) scoring (Fig. 1C) further demonstrated distinct dysregulation patterns, and the top 10 VIP-ranked metabolites were additionally visualized in a bar chart (Fig. 1D). Notably, an exploratory volcano plot identified LPC 18:2 as the most markedly decreased lipid species (Fig. 1E), a finding bolstered by its significant inverse correlation with BMI across the entire asthma cohort (Fig. 1F). By contrast, serum LPC 18:2 levels were not significantly correlated with ACT score, blood eosinophil counts, total IgE, FeNO, FEV₁% predicted, or FEV₁/FVC (all *P* > 0.05; Supplementary Table S1). 

To further clarify the clinical correlates of LPC 18:2 dysregulation, we examined its distribution across predefined asthma subgroups. Patients with obese asthma exhibited significantly lower serum LPC 18:2 levels than those with non-obese asthma (*P* = 0.001). When patients were stratified by asthma control status, LPC 18:2 concentrations were lower in those with uncontrolled asthma than in those with controlled disease (*P* = 0.005). A similar pattern was observed with respect to treatment intensity, with patients receiving GINA Step 5 therapy displaying reduced LPC 18:2 levels compared with those on Steps 1–4 (*P* = 0.003). Among type 2–related biomarkers, LPC 18:2 levels were higher in patients with high FeNO than in those with low FeNO (*P* = 0.041), whereas no significant difference was detected between eosinophil-high and eosinophil-low groups (*P* = 0.149). All subgroup comparisons are shown in Fig. 1G. Stratification by FEV₁% predicted (≥ 80% vs. < 80%) and FEV₁/FVC (≥ 70% vs. < 70%) likewise did not reveal significant differences in LPC 18:2 levels (both *P* > 0.05; Supplementary Fig. S2). Taken together, these subgroup analyses suggest that reduced LPC 18:2 is more frequently observed in obese, poorly controlled asthma at the more severe end of the treatment spectrum, rather than simply mirroring airflow limitation.

We next sought to independently validate this discovery in a murine model of obese asthma. PLS-DA again showed distinct clustering between obese and non-obese asthmatic mice (Fig. 1H). Consistent with the human data, LPC 18:2 was highlighted as a key discriminatory lipid by VIP analysis (Fig. 1I). In line with this, volcano plot analysis under stringent statistical criteria (FDR–adjusted *P* < 0.05 and |fold change| > 2.0) confirmed a conserved decrease of LPC 18:2 in the obese group (Fig. 1J). This cross-species consistency supports LPC 18:2 downregulation as a conserved metabolic feature of obese asthma and implicates it as a candidate metabolic mediator in disease pathogenesis, prompting us to examine its functional impact on airway inflammation and T helper cell polarization in subsequent experiments.

### Obesity exacerbates airway pathology in a murine asthma model

**Fig. 2:**
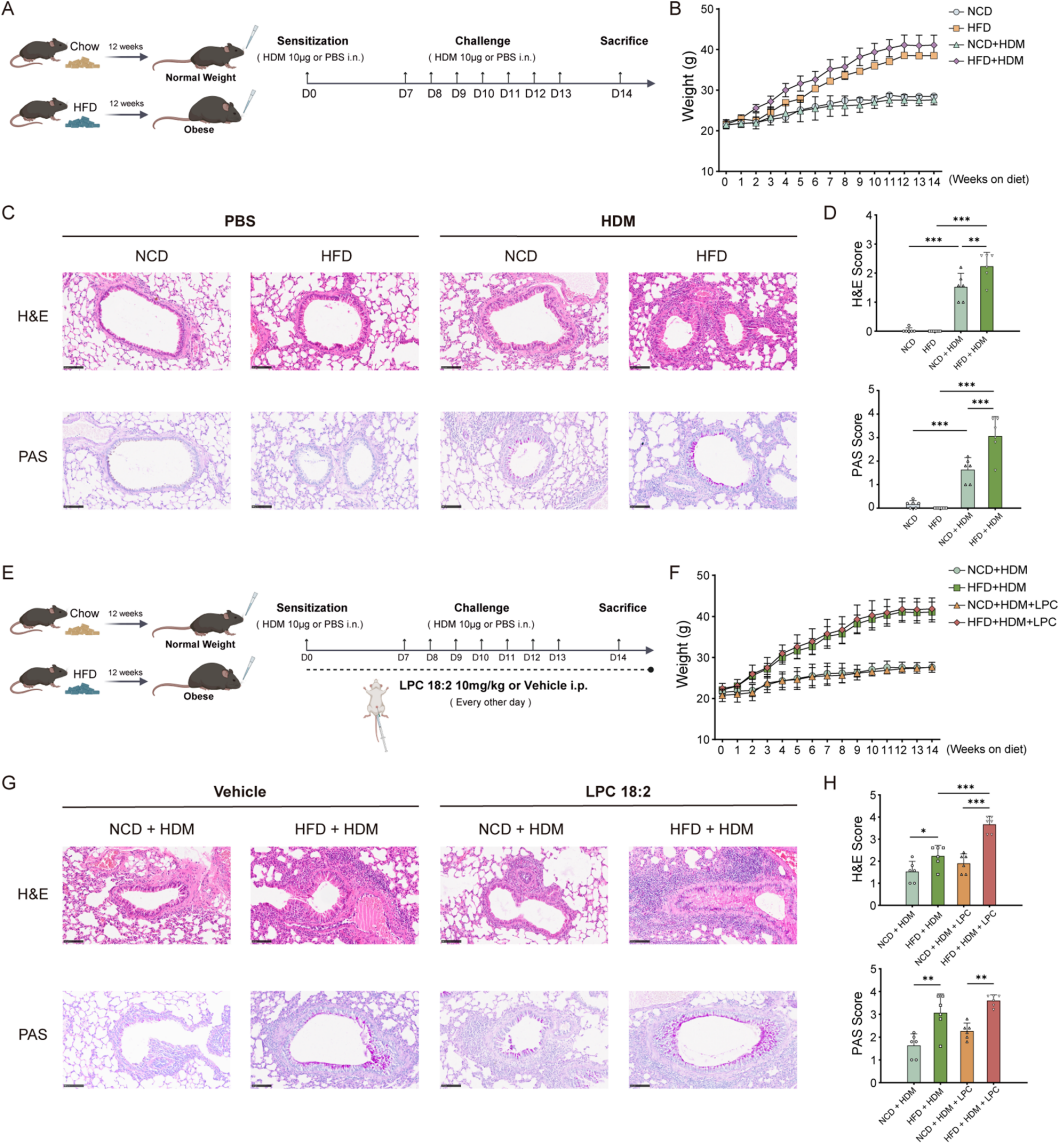
Obesity aggravates airway inflammation in a murine asthma model, and LPC 18:2 further amplifies this inflammatory response. **A** Schematic illustration of the experimental protocol. 6-week-old male C57BL/6J mice were fed a normal chow diet (NCD) or a high-fat diet (HFD) for 12 weeks, followed by intranasal sensitization and challenge with HDM extract. **B** Body weight trajectories during the 12-week diet period and subsequent HDM-induced asthma phase. **C** Representative H&E- and PAS-stained lung sections from the NCD, HFD, NCD + HDM and HFD + HDM groups (scale bar = 100 µm). **D** Quantification of airway inflammation and mucus production corresponding to panel **C**. **E** Schematic illustration of LPC 18:2 administration. NCD + HDM and HFD + HDM mice received intraperitoneal injections of LPC 18:2 or vehicle every other day during asthma modeling (days 1–13; for a total of seven injections). **F** Body weight trajectories during diet feeding and the HDM-induced asthma phase in mice treated with LPC 18:2 or vehicle. **G** Representative H&E- and PAS-stained lung sections from the NCD + HDM, HFD + HDM, NCD + HDM + LPC and HFD + HDM + LPC groups (scale bar = 100 μm). **H** Quantification of airway inflammation and mucus production corresponding to panel **G**. Data are presented as mean ± SD (*n* = 6 mice per group). Statistical analyses were performed using one-way ANOVA followed by Tukey’s post hoc test. *P < 0.05, **P < 0.01, ***P < 0.001

To examine the impact of obesity on asthma pathogenesis, 6-week-old male C57BL/6J mice were fed either a normal chow diet (NCD) or a high-fat diet (HFD) for 12 weeks, followed by sensitization and challenge with HDM extract (Fig. 2A). HFD-fed mice gained substantial weight (≥ 20% compared with NCD-fed controls) by week 12, confirming successful induction of obesity (Fig. 2B). HDM exposure induced peribronchial inflammation and goblet cell hyperplasia in both diet groups, validating the asthma model. However, HFD-fed asthmatic mice exhibited markedly greater airway inflammation, enhanced goblet cell hyperplasia, and increased mucus accumulation compared with NCD-fed asthmatic mice **(**Fig. 2C**)**. Histological assessment and quantitative scoring corroborated these observations (Fig. 2D), indicating that obesity substantially aggravates asthma-associated airway pathology.

### LPC 18:2 administration amplifies airway inflammation in obese asthmatic mice

Given the marked reduction of circulating LPC 18:2 in obese asthma, we therefore tested whether restoring LPC 18:2 could modulate airway inflammation in vivo by administering exogenous LPC 18:2. Starting at week 13, asthmatic mice received intraperitoneal injections of LPC 18:2 every other day for 14 days (for a total of seven doses; Fig. 2E). Body weight remained stable throughout the treatment period (Fig. 2F). In obese asthmatic mice, LPC 18:2 administration significantly increased histopathological inflammation, as reflected by higher H&E scores (Fig. 2G), whereas mucus production, assessed by PAS staining, remained unchanged (Fig. 2H). By contrast, LPC 18:2 treatment induced only minimal, non-significant histopathological changes in normal-weight asthmatic mice. Taken together, these findings suggest a model in which exogenous LPC 18:2 acts as an obesity-dependent amplifier of airway inflammation in asthma, with its pro-inflammatory effects most prominently expressed in the obese asthmatic airway.

### Obesity promotes a shift toward Th17-polarized immune responses in experimental asthma

**Fig. 3:**
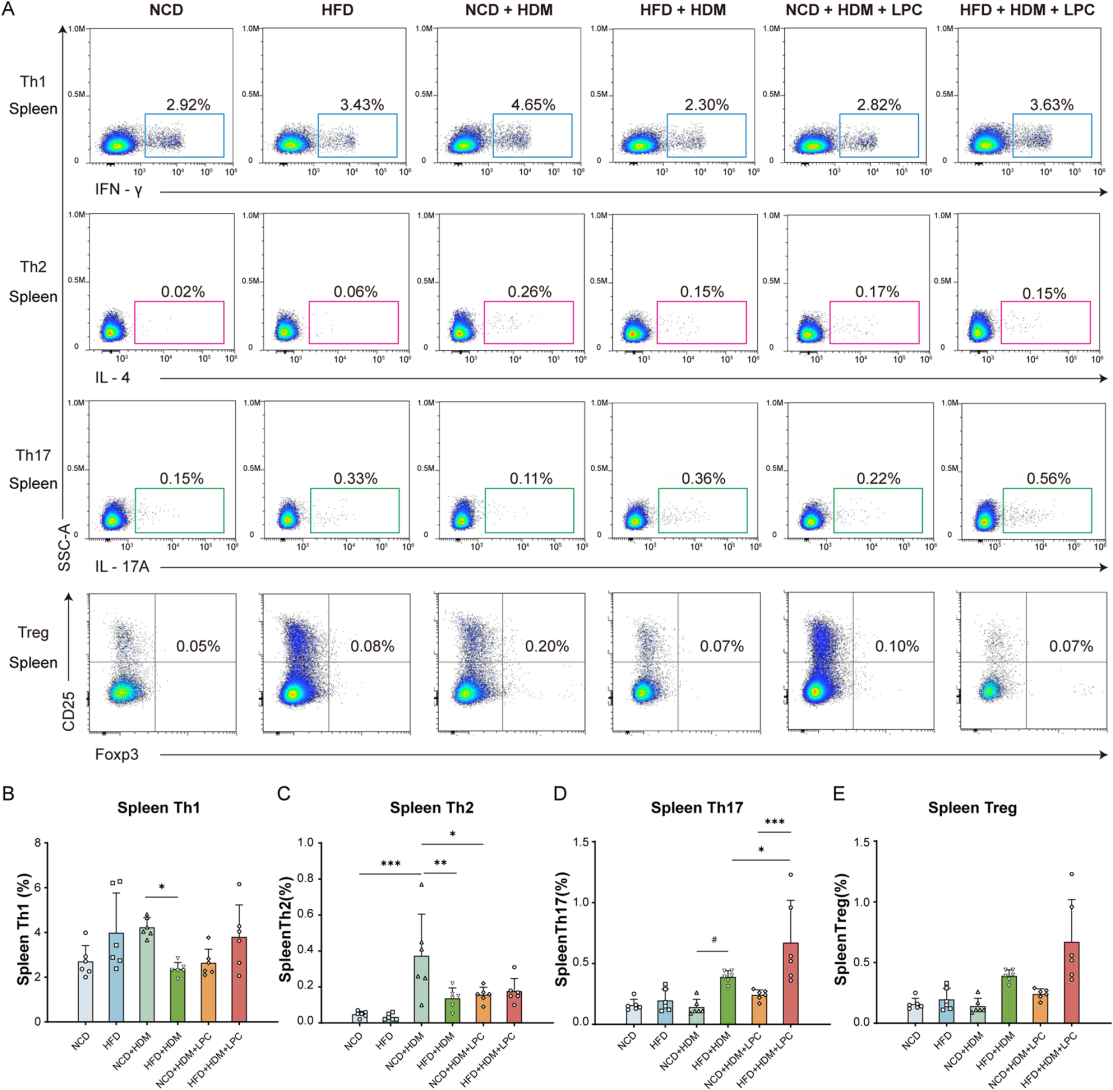
LPC 18:2 enhances Th17 responses in the spleen of obese asthmatic mice. **A** Representative flow cytometric gating strategy used to identify splenic Th1, Th2, Th17, and Treg subsets. **B**–**E** Quantification of splenic Th1 (**B**), Th2 (**C**), Th17 (**D**), and Treg (**E**) frequencies across the six experimental groups. Data are presented as mean ± SD (*n* = 6 mice per group). Statistical analyses were performed using one-way ANOVA followed by Tukey’s post hoc test. *P < 0.05, **P < 0.01, ***P < 0.001, #*P* = 0.05–0.10

**Fig. 4:**
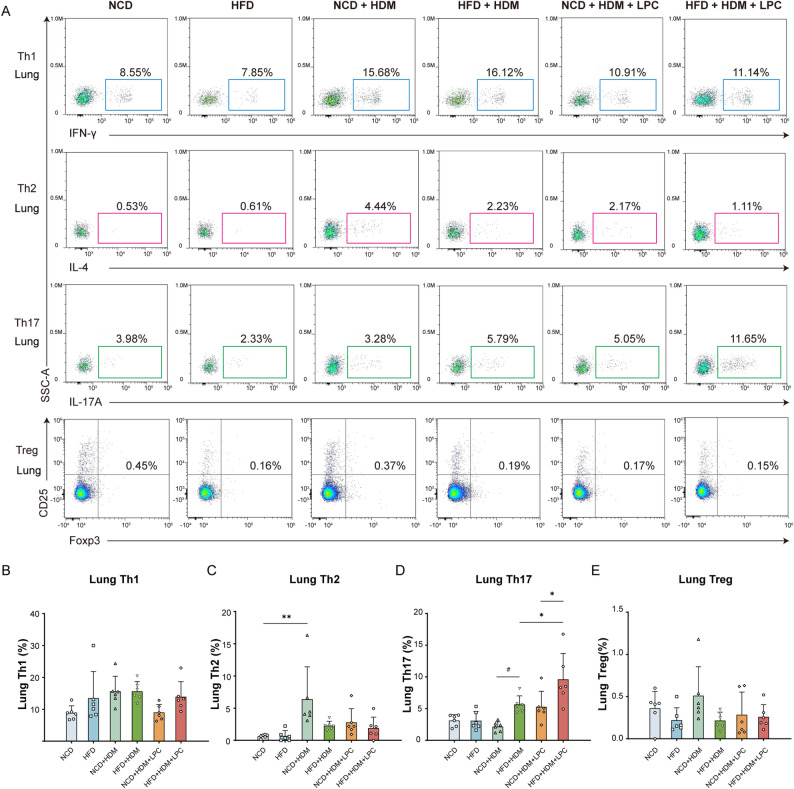
LPC 18:2 enhances Th17 responses in the lungs of obese asthmatic mice. **A** Representative flow cytometric gating strategy used to identify pulmonary CD4⁺ Th1, Th2, Th17, and Treg subsets in lung single-cell suspensions. **B**–**E** Quantification of pulmonary Th1 (**B**), Th2 (**C**), Th17 (**D**), and Treg (**E**) frequencies across the six experimental groups. Data are presented as mean ± SD (*n* = 6 mice per group). Statistical analyses were performed using one-way ANOVA followed by Tukey’s post hoc test. *P < 0.05, **P < 0.01, ***P < 0.001, #*P* = 0.05–0.10

To assess T cell polarization in obese asthma, we performed flow cytometric profiling of splenic and pulmonary CD4⁺ T-cell subsets. In the spleen (Fig. 3A), obese asthmatic mice exhibited significant reductions in Th1 (*P* = 0.040; Fig. 3B) and Th2 (*P* = 0.005; Fig. 3C) frequencies compared with normal-weight asthmatic controls. In contrast, Th17 cells showed an upward trend (*P* = 0.081; Fig. 3D), whereas Treg frequencies were unaltered (*P* > 0.05; Fig. 3E). In the lungs (Fig. 4A), the frequencies of Th1 and Th2 cells were comparable between obese and normal-weight asthmatic mice (*P* > 0.05; Fig. 4B, C). Consistent with the splenic data, pulmonary Th17 cells tended to be more frequent in obese asthmatic mice (*P* = 0.088; Fig. 4D), while Treg frequencies again remained unchanged (*P* > 0.05; Fig. 4E). Although the increase in Th17 cells did not reach conventional statistical significance, the concordant upward trends in both spleen and lung, together with the marked reductions in splenic Th1 and Th2 subsets, were consistent with a shift in CD4⁺ T-cell polarization toward a Th17-prone phenotype in obese asthmatic mice.

### LPC 18:2 selectively enhances Th17 responses in obese asthmatic mice

Because obese asthma in our model was associated with both a tendency toward increased Th17 polarization and reduced circulating LPC 18:2 levels, we next investigated whether LPC 18:2 could contribute to Th17-associated inflammation in vivo. In obese asthmatic mice, LPC 18:2 treatment significantly increased Th17 frequencies in both the spleen (from 0.39% ± 0.05% to 0.67% ± 0.35%, *P* = 0.035; Fig. 3D) and the lungs (from 5.66 ± 1.35% to 9.61 ± 4.11%, *P* = 0.039; Fig. 4D), consistent with enhanced Th17 responses at both systemic and pulmonary sites. In contrast, LPC 18:2 administration had little effect on Th17 frequencies in normal-weight asthmatic mice (*P* > 0.05). Among LPC-treated animals, obese asthmatic mice exhibited significantly higher Th17 frequencies than their normal-weight counterparts in both the spleen (*P* < 0.001; Fig. 3D) and the lungs (*P* = 0.019; Fig. 4D), whereas splenic Th1, Th2, and Treg frequencies remained unchanged (Fig. 3B, C and E). Similarly, no differences were observed in pulmonary Th1, Th2, or Treg frequencies (Fig. 4B, C and E). Together, these findings suggest that LPC 18:2 preferentially amplifies Th17 responses in the context of obesity, raising the possibility that obesity-associated alterations in lipid metabolism may potentiate LPC-related immune activation and thereby contribute to airway inflammation, consistent with our histopathological findings.

### LPC 18:2 dose-dependently promotes Th17 polarization in vitro

**Fig. 5:**
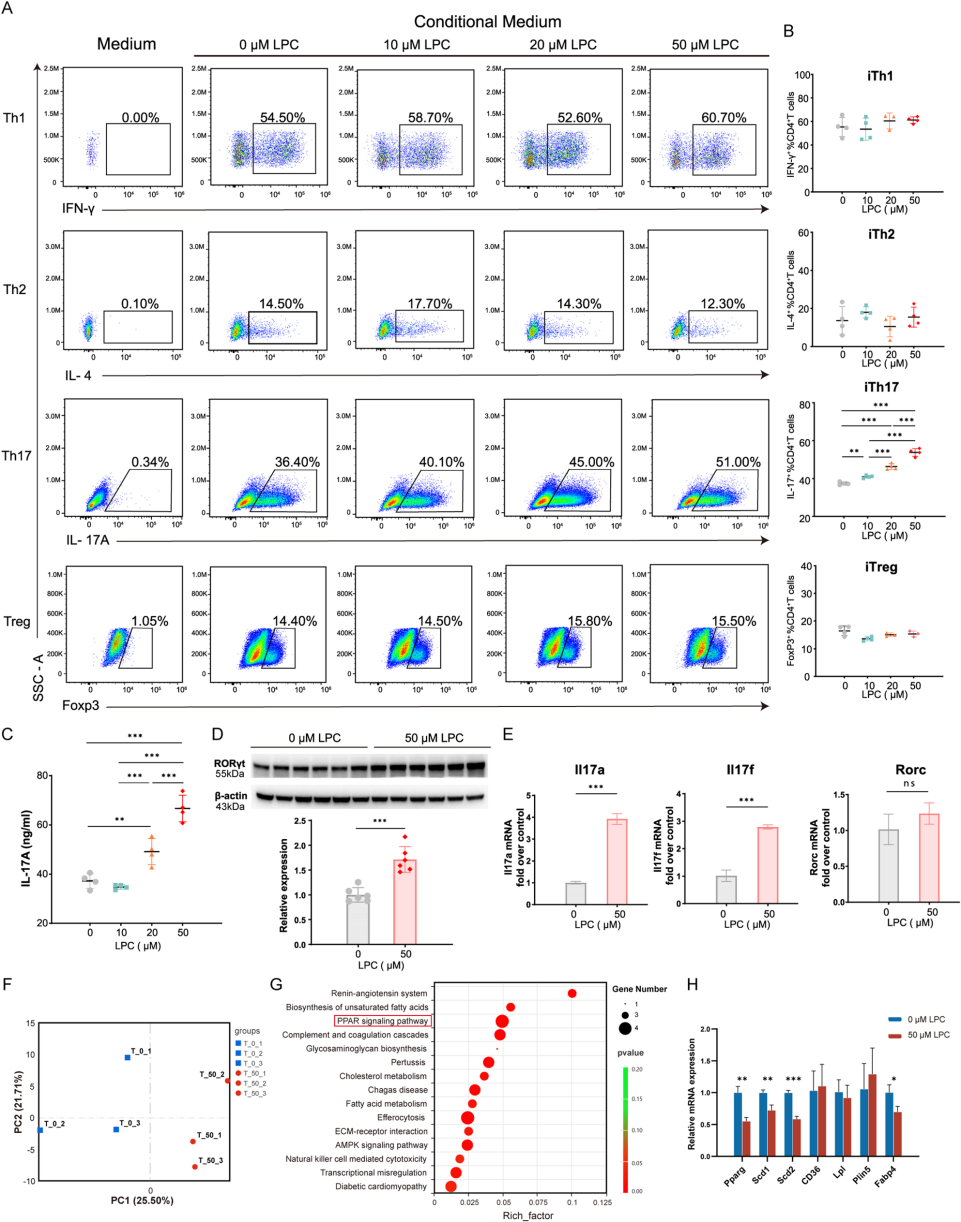
LPC 18:2 promotes Th17 differentiation in vitro. **A** Naïve CD4⁺ T cells were isolated from mouse spleens and stimulated with anti-CD3 and anti-CD28 antibodies under neutral, Th1, Th2, Th17, or Treg-polarizing conditions in the presence of increasing concentrations of LPC 18:2 (0, 10, 20, or 50 µM) for 72 h. Differentiation into IFN-γ⁺ Th1, IL-4⁺ Th2, IL-17A⁺ Th17, and Foxp3⁺ Treg cells was assessed by flow cytometry. **B** Quantification of CD4⁺ T-cell subset frequencies under different LPC 18:2 concentrations. **C** IL-17A secretion in culture supernatants of induced Th17 (iTh17) cells after 72 h of differentiation. **D** RORγt protein expression in iTh17 cells treated with 0 or 50 µM LPC 18:2, assessed by Western blot (uncropped membrane scans are shown in Additional file 5). **E** Relative mRNA expression of *Il17a*, *Il17f*, and *Rorc* in iTh17 cells treated with 0 or 50 µM LPC 18:2, measured by RT–qPCR. **F** Principal component analysis (PCA) showing separation of LPC 18:2–treated and control iTh17 cells. **G** KEGG pathway enrichment analysis of differentially expressed genes highlighting enrichment of the PPAR signaling pathway. **H** RT–qPCR analysis of *Pparg* and its downstream target genes (*Scd1*, *Scd2*, *Cd36*, *Lpl*, *Plin5*, *Fabp4*) in iTh17 cells treated with 0 or 50 µM LPC 18:2. Data are presented as mean ± SD. Statistical analyses were performed using one-way ANOVA with Tukey’s post hoc test for multi-group comparisons and an unpaired two-tailed *t*-test for two-group comparisons, as appropriate. *P < 0.05, **P < 0.01, ***P < 0.001. ns, not significant

To explore the cellular mechanisms underlying the Th17-enhancing effects of LPC 18:2 observed in vivo, we performed in vitro differentiation assays using naïve CD4^+^ T cells purified from mouse spleens. Magnetic bead isolation yielded > 90% purity in all cases (Supplementary Fig. S3). Cells were activated with anti-CD3 and anti-CD28 and cultured under Th1, Th2, Th17, or Treg-polarizing conditions in the presence of increasing concentrations of LPC 18:2 (0, 10, 20, and 50 µM) **(**Fig. 5A**)**. LPC 18:2 selectively promoted Th17 differentiation in a dose-dependent manner, as evidenced by elevated frequencies of IL-17A⁺CD4⁺ T cells **(**Fig. 5B**)**, increased IL-17A secretion **(**Fig. 5C**)** and upregulation of the Th17 lineage-defining transcription factor ROR**γ**t **(**Fig. 5D**)**. Consistently, LPC 18:2 markedly increased *Il17a* and *Il17f* mRNA expression and showed a trend toward higher *Rorc* levels, although the latter did not reach statistical significance **(**Fig. 5E**)**. LPC 18:2 had no detectable effect on Th1, Th2, or Treg differentiation. Importantly, LPC 18:2 did not affect Th17 viability or apoptosis (Supplementary Fig. S4), indicating a specific effect on lineage commitment rather than cell survival or expansion.

To further define the molecular basis of LPC 18:2–induced Th17 differentiation, we performed RNA sequencing (RNA-seq) on naïve CD4⁺ T cells differentiated under Th17-polarizing conditions in the presence or absence of LPC 18:2 (50 µM). Principal component analysis (PCA) showed segregation between LPC 18:2-treated and control Th17 cells, indicating a transcriptional response to LPC exposure (Fig. 5F). KEGG pathway enrichment analysis of differentially expressed genes highlighted lipid metabolism–related pathways, with the PPAR signaling pathway emerging as the most significantly enriched (Fig. 5G). Guided by these findings, we focused subsequent validation on the PPARγ pathway. Quantitative RT–PCR confirmed reduced expression of *Pparg* (encoding PPARγ) and key downstream targets involved in fatty acid metabolism (*Scd1*, *Scd2*, and *Fabp4*) in LPC 18:2-treated Th17 cells compared with controls (Fig. 5H). Collectively, these results suggest that LPC 18:2 is associated with transcriptional downregulation of PPARγ-related lipid metabolic programs in differentiating Th17 cells, suggesting a potential link between LPC 18:2–responsive lipid metabolism and Th17 cell programming.

### Enhanced cellular LPC uptake may help explain the paradox of reduced circulating levels in obese asthma

Although LPC 18:2 amplified Th17 responses and airway inflammation in obese asthma, its circulating levels were paradoxically reduced. One potential explanation could be increased conversion of LPC to LPA via autotaxin. To evaluate this possibility, we quantified autotaxin and LPA species in both patients and mice. In the human cohort, plasma autotaxin concentrations were numerically higher in obese asthma than in normal-weight asthma, but did not reach statistical significance (614.78 ± 215.16 ng/mL vs. 477.41 ± 270.12 ng/mL; *P* = 0.095; Supplementary Fig. S5A). In the murine HDM-induced obese asthma model, autotaxin levels in lung homogenates and bronchoalveolar lavage fluid (BALF) were comparable between obese and non-obese asthmatic mice (both *P* > 0.05; Supplementary Fig. S5B, C), and plasma autotaxin concentrations were below the limit of detection in both groups. Consistent with protein-level measurements, plasma autotaxin lysoPLD activity assessed by a fluorogenic kinetic assay was comparable between normal-weight and overweight/obese asthma (382.68 ± 108.74 vs. 377.14 ± 108.30 ATX units; *P* = 0.873; Supplementary Fig. S5D).

We next profiled individual LPA species in both patients and mice. In the human cohort, serum LPA species showed broadly similar levels in patients with obese asthma versus normal-weight asthma, and no individual LPA species reached the predefined thresholds for differential abundance in exploratory analyses (Supplementary Fig. S5E, F). In the murine model, obese asthmatic animals showed a mild but significant reduction in serum LPA 18:2 compared with non-obese asthmatic controls (*P* = 0.021). Other LPA species did not exhibit a consistent pattern of change and none met the significance criteria in confirmatory analyses (Supplementary Fig. S5G, H). Together with the observed reduction in LPC 18:2, these data indicate that reduced LPC 18:2 in obese asthma is not accompanied by a compensatory rise in circulating LPA 18:2 in either patients or mice, suggesting that systemic autotaxin-driven conversion of LPC 18:2 to LPA 18:2 is unlikely to be the sole or predominant explanation for its reduced circulating levels.

In complementary in vitro experiments, we further examined whether pharmacological inhibition of the autotaxin–LPA axis modifies the Th17-promoting effect of LPC 18:2. Naïve CD4⁺ T cells were differentiated under Th17-polarizing conditions in the presence or absence of LPC 18:2 (50 µM), together with the autotaxin inhibitor PF-8380 (0.1 or 1 µM). As expected, LPC 18:2 increased the frequency of IL-17A⁺ CD4⁺ T cells compared with vehicle-treated iTh17 cultures, and this increase was not attenuated in the presence of PF-8380 (Supplementary Fig. S6A, B). Because LPA signals through six G protein–coupled receptors (LPA₁–₆) and selective small-molecule antagonists are currently available for LPA₁–₃, we next targeted LPA₁–₃ using a receptor antagonist cocktail. Naïve CD4⁺ T cells were cultured under the same iTh17 conditions with or without LPC 18:2 (50 µM), in the presence of Ki16425 (an LPA₁/₃ antagonist) and H2L5186303 (an LPA₂ antagonist) at 1 or 5 µM each. Under these conditions, LPC 18:2 again increased IL-17A⁺ CD4⁺ T-cell frequencies relative to control cultures, and this enhancement was preserved despite LPA₁–₃ receptor blockade (Supplementary Fig. S6C, D). Together, these data suggest that the Th17-promoting effect of LPC 18:2 in this in vitro setting is not abolished by acute inhibition of either autotaxin or LPA₁–₃ receptor signaling.

**Fig. 6:**
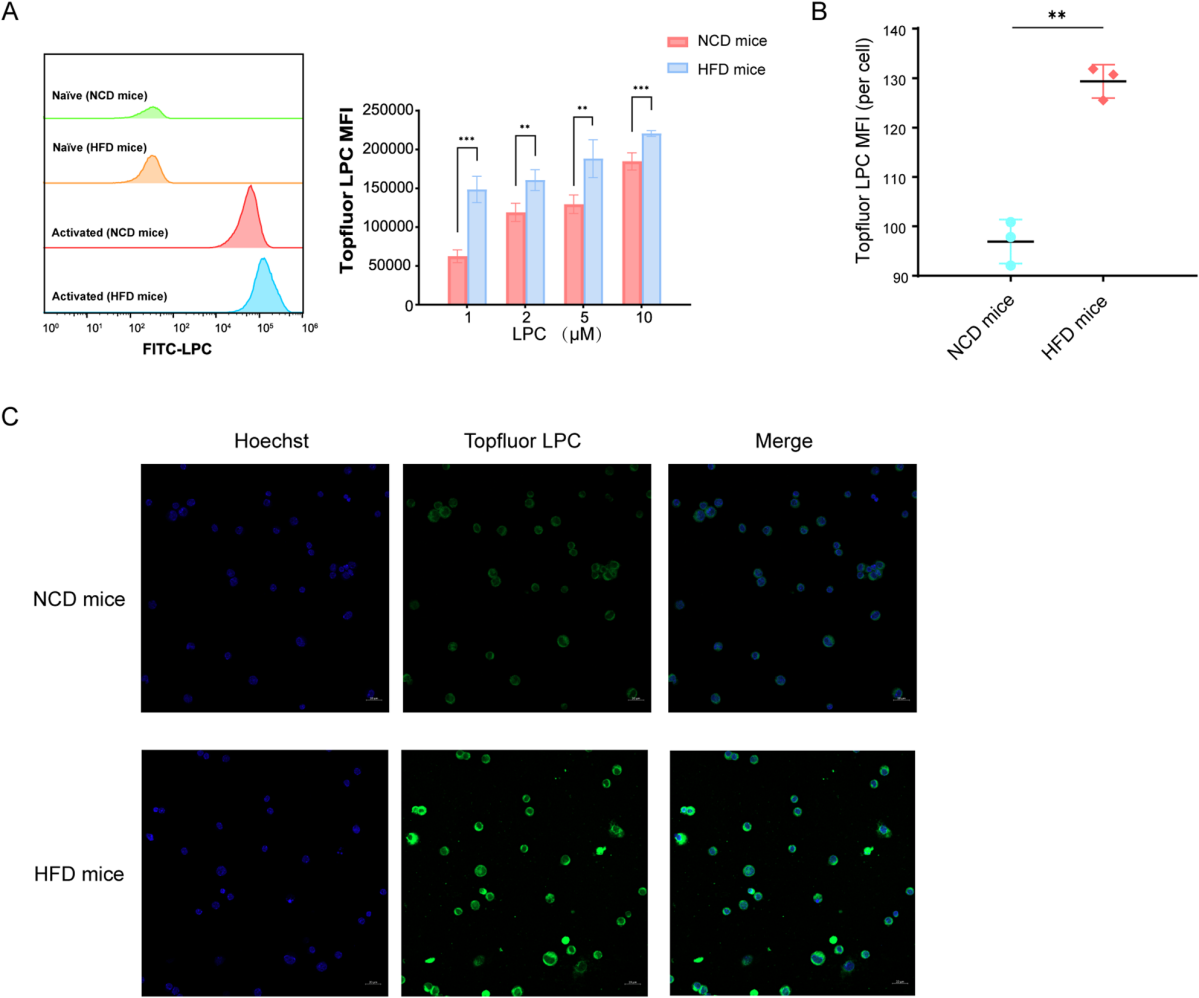
Enhanced LPC uptake by naïve CD4⁺ T cells from obese mice. **A** Flow cytometric quantification of TopFluor–LPC uptake (MFI) in naïve splenic CD4⁺ T cells following TCR stimulation with anti-CD3 and anti-CD28, across increasing TopFluor–LPC concentrations (1, 2, 5, and 10 µM). **B** Quantification of TopFluor–LPC MFI per naïve CD4⁺ T cell by confocal microscopy in NCD- and HFD-fed mice. **C** Representative confocal images showing increased TopFluor–LPC signal (green) in naïve CD4⁺ T cells from HFD mice; nuclei were stained with Hoechst (blue). Scale bar = 20 µm. Data are presented as mean ± SD (*n* = 3 mice per group). Statistical analyses were performed using two-way repeated-measures ANOVA with Tukey’s post hoc test for panel A and an unpaired two-tailed *t*-test for panel B. *P < 0.05, **P < 0.01, ***P < 0.001

We therefore explored whether enhanced cellular uptake might instead contribute to the paradox of reduced circulating LPC 18:2 levels despite its pro-inflammatory effects. Using TopFluor–LPC, we found that TCR stimulation with anti-CD3 and anti-CD28 markedly augmented LPC uptake by naïve splenic CD4⁺ T cells compared with unstimulated controls. Notably, naïve CD4⁺ T cells from obese mice consistently exhibited higher uptake than those from lean mice across all tested concentrations (1, 2, 5, and 10 µM), with fluorescence intensity increasing dose-dependently in both groups (Fig. 6A). Confocal microscopy corroborated these results, with quantitative image analysis revealing significantly higher mean TopFluor intensity per cell in obese versus lean naïve CD4⁺ T cells (Fig. 6B). Representative confocal images further illustrate the enhanced intracellular signal in the obese group (Fig. 6C). These findings suggest that obesity may promote enhanced LPC acquisition by naïve CD4⁺ T cells, a mechanism that may account for both the reduced circulating LPC 18:2 levels and its heightened pro-inflammatory activity in obese asthma. Targeting LPC uptake pathways—such as LPC transporters and their regulatory networks—may therefore represent a potential strategy to rebalance lipid–immune crosstalk in obese asthma.

## Discussion

In this study, we integrated targeted lipidomics, clinical phenotyping, and mechanistic experiments in mice and primary T cells to investigate how obesity-associated lipid disturbances contribute to asthma pathobiology. We identified LPC 18:2 as a consistently downregulated glycerophospholipid in obese asthma across human and murine models, and showed that exogenous LPC 18:2 preferentially exacerbates Th17-skewed airway inflammation and pathology in obese, but not lean, asthmatic mice. In vitro, LPC 18:2 selectively promoted Th17 differentiation without affecting Th1, Th2, or Treg lineages and was associated with transcriptional downregulation of PPARγ-related lipid metabolic pathways in differentiating Th17 cells. Mechanistically, we found no evidence for a compensatory increase in circulating LPA species or for a major contribution of systemic autotaxin–LPA conversion in explaining reduced LPC 18:2 levels. Instead, naïve CD4⁺ T cells from obese mice exhibited enhanced uptake of LPC, suggesting that increased cellular acquisition and utilization of LPC 18:2 may contribute to its decreased circulating abundance and accentuated immunologic impact in obese asthma.

Our observation of reduced circulating LPC 18:2 aligns with population-based lipidomic studies showing an inverse association between total LPC abundance and BMI [28, 29] and reports demonstrating decreased levels of multiple LPC species in obese adolescents and adults [30, 31]. These studies collectively suggest that systemic LPC depletion is a characteristic feature of obesity-related metabolic disturbance, although they primarily describe associations with obesity per se and do not address functional roles in adaptive immunity relevant to asthma. Our findings extend this literature by demonstrating that obesity-associated depletion of LPC 18:2 occurs in asthma and is functionally linked to enhanced Th17 differentiation, at least in our experimental systems, particularly in the context of obesity. More broadly, prior reports have highlighted that LPC species can exert divergent, even opposing, effects across diseases, promoting inflammation in some settings [32–34] while appearing protective in others [35, 36]. These observations suggest that the immunologic outcome of LPC exposure depends not only on its abundance and molecular species but also on the cellular and metabolic context. Our finding that LPC 18:2 selectively enhances Th17 responses in obese, but not lean, asthmatic mice reinforces this concept.

Within our asthma cohort, serum LPC 18:2 showed a discernible clinical pattern. Levels were lower in obese than in non-obese asthma and were similarly reduced in patients with uncontrolled disease and in those receiving GINA Step 5 therapy compared with individuals on less intensive treatment, indicating that low LPC 18:2 tends to cluster in a more severe, poorly controlled subset. By contrast, with respect to type 2–related markers, subgroup analyses indicated that LPC 18:2 levels were relatively higher in patients with elevated FeNO, whereas no clear differences were observed between eosinophil-high and eosinophil-low groups, consistent with the lack of significant correlations between LPC 18:2 and blood eosinophils, total IgE, or FeNO in the full cohort. Together, these observations suggest that low LPC 18:2 is more characteristic of an obese, poorly controlled asthma phenotype than of classical type 2–high inflammation. This pattern is consistent with the possibility that LPC 18:2 may reflect an obesity-associated, non–type 2–biased endotype, a hypothesis that will require confirmation in larger and more deeply phenotyped populations.

Beyond systemic lipid alterations, our HDM-induced asthma model demonstrates that obesity aggravates allergic airway disease at the tissue and immune levels. Obese asthmatic mice developed more pronounced peribronchial inflammation, goblet cell hyperplasia, and mucus accumulation than their lean counterparts, accompanied by a shift in CD4⁺ T-cell polarization characterized by reduced Th1/Th2 frequencies and a trend toward increased Th17 cells in both lung and spleen. These observations are consistent with reports from other inflammatory settings in which obesity promotes Th17 expansion and worsens disease severity, including models of inflammatory bowel disease [37], atopic dermatitis [13], and airway hyperresponsiveness [38]. Within this Th17-biased, obesity-conditioned landscape, our functional experiments indicate that LPC 18:2 acts as a context-dependent amplifier of inflammation. In obese asthmatic mice, LPC 18:2 administration further exacerbated airway inflammation and selectively increased Th17 frequencies in the spleen and lungs, whereas Th17 responses in lean asthmatic mice were minimally affected. Together, these findings support the view that obesity creates an immune–metabolic milieu in which LPC 18:2 preferentially drives Th17-skewed inflammation, in line with emerging clinical and experimental evidence implicating IL-17–driven pathways in non–type 2 and steroid-insensitive asthma, particularly in obese individuals.

Our in vitro differentiation assays further support a direct, lineage-selective effect of LPC 18:2 on Th17 cells. Under Th17-polarizing conditions, LPC 18:2 dose-dependently increased the frequency of IL-17A⁺ CD4⁺ T cells and IL-17A secretion, without detectable effects on Th1, Th2, or Treg differentiation, indicating a lineage-specific effect rather than a broad amplification of inflammation. At the transcriptional level, RNA sequencing of LPC 18:2–treated Th17 cells highlighted enrichment of lipid metabolism–related pathways, with PPAR signaling emerging as the most significantly affected pathway. PPARγ has been identified as an intrinsic negative regulator of Th17 differentiation [39–43], in part by constraining RORγt activity and counteracting pro-inflammatory transcription factors such as NF-κB, AP-1, and STAT3 [44, 45]. Guided by these results, we validated reduced expression of *Pparg* and several PPARγ target genes involved in fatty acid processing (such as *Scd1*, *Scd2*, and *Fabp4*) in LPC 18:2–exposed Th17 cells by quantitative RT–qPCR. Although we did not perform protein-level or rescue experiments to establish a causal role for PPARγ, these transcriptional changes suggest that LPC 18:2 exposure is associated with remodeling of PPARγ-dependent lipid metabolic programs in Th17 cells. In light of the established role of PPARγ as an intrinsic brake on Th17 differentiation, this pattern is consistent with reduced PPARγ-mediated restraint and may help explain the propensity toward Th17 skewing in the obesogenic context.

A notable feature of our data is that obese asthmatic mice exhibit reduced circulating LPC 18:2 yet show an exaggerated response to exogenous LPC 18:2. Given that LPC can be converted to LPA by autotaxin, a secreted lysophospholipase D that hydrolyzes LPC to LPA and choline [46], and that LPA then signals through G protein–coupled LPA₁–₆ receptors on immune and structural cells [47], this axis represents a plausible route for LPC catabolism and immunomodulation. We therefore first considered whether enhanced autotaxin–LPA activity might help account for LPC 18:2 depletion in obese asthma. However, as detailed in the Results, we did not find clear evidence for systemic upregulation of the autotaxin–LPA axis in either patients or mice. In complementary in vitro experiments, pharmacological inhibition of autotaxin with PF-8380 and combined blockade of LPA₁–₃ receptors did not abolish the Th17-promoting effect of LPC 18:2 in iTh17 cultures. While these findings do not exclude more localized or chronic contributions of autotaxin–LPA signaling in vivo, they suggest that acute systemic activation of this pathway is unlikely to be the predominant driver of reduced circulating LPC 18:2 nor of its Th17-skewing effects in our models. Instead, our uptake studies point to enhanced cellular acquisition of LPC as a plausible contributing mechanism. We found that TCR stimulation markedly increased uptake of fluorescent LPC by naïve CD4⁺ T cells, and that naïve CD4⁺ T cells from obese mice consistently internalized more LPC than those from lean mice across all tested concentrations. These observations support a model in which obesity-associated changes in T-cell lipid handling increase LPC uptake, thereby lowering circulating LPC 18:2 levels and amplifying its local impact on Th17 differentiation within lymphoid and airway tissues. In this framework, LPC uptake pathways emerge as potential targets for modulating lipid–immune crosstalk in obese asthma. While the specific mechanisms underlying this altered uptake remain undefined, plausible contributors include changes in lipid transporters, membrane composition, or intracellular remodeling pathways. Future studies will aim to delineate the molecular machinery governing LPC uptake and to determine how obesogenic metabolic cues sensitize T cells to LPC-driven Th17 polarization.

## Conclusions

In conclusion, our findings support a translational framework in which obesity-related alterations in lipid metabolism intersect with T-cell differentiation to promote a Th17-skewed, non–type 2–biased asthma endotype. LPC 18:2 emerges not merely as a passive biomarker, but rather as a candidate metabolic mediator that is more frequently reduced in obese, poorly controlled asthma, exerts direct Th17-promoting effects in vitro, and exacerbates airway inflammation in obese asthmatic mice. Mechanistically, our findings are compatible with the possibility that obesity-associated changes in T-cell lipid handling, including enhanced LPC uptake and remodeling of PPARγ-related lipid metabolic programs, may help explain reduced circulating LPC 18:2 and its Th17-skewing effects in obese asthma.

These conclusions should be interpreted in light of several limitations, including the modest sample size of the single-center human cohort, the cross-sectional design, the targeted rather than global lipidomic coverage, and the absence of direct genetic or pharmacologic manipulation of PPARγ or LPC transport pathways. Future work in larger, multicenter cohorts, together with mechanistic studies dissecting the LPC–PPARγ–Th17 axis and the molecular machinery governing LPC 18:2 uptake, will be important to validate and refine this model and to determine whether therapeutic modulation of lipid metabolism can be leveraged to improve outcomes in obese asthma.

## Supplementary Information

Additional file 1: Human serum lipidomics dataset. This spreadsheet contains the lipidomics data obtained from human serum samples.

Additional file 2: Mouse serum lipidomics dataset. This spreadsheet contains the lipidomics data obtained from mouse serum samples.

Additional file 3: Supplementary Methods. This file provides comprehensive and step-by-step protocols for the additional experimental methodologies referenced in the manuscript.

Additional file 4: Supplementary Figures S1–S6 and Supplementary Table S1. Figure S1. presents FMO and isotype controls used for flow cytometry gating of CD4⁺ T cell subsets. Figure S2. shows serum LPC 18:2 levels stratified by pre-bronchodilator FEV₁% predicted and FEV₁/FVC in asthma patients. Figure S3. documents the flow cytometric validation of naïve CD4⁺ T cell isolation from mouse spleens. Figure S4. evaluates the effects of LPC 18:2 on cell viability and apoptosis in iTh17 cells. Figure S5. summarizes autotaxin and LPA profiles in obese versus non-obese asthma in human and murine samples. Figure S6. illustrates the impact of pharmacological inhibition of the autotaxin–LPA axis on LPC 18:2–induced Th17 differentiation in vitro. Supplementary Table S1 presents correlation analyses of LPC 18:2 levels with asthma-related parameters.

Additional file 5: Supplementary Figure S7. This file contains the original, uncropped Western blot membranes for RORγt and β-actin corresponding to Fig. 5D.

## Data Availability

The RNA-seq data generated in this study have been deposited in the NCBI Sequence Read Archive (SRA) under BioProject accession PRJNA1344896. Targeted lipidomic datasets are provided in the supplementary materials (Additional files 1 and 2). Additional data supporting the findings of this study are available from the corresponding author upon reasonable request.

## Abbreviations

ACT: Asthma Control Test
ATX: Autotaxin
BALF: Bronchoalveolar lavage fluid
BEC: Blood eosinophil count
BFA: Brefeldin A
BMI: Body mass index
CUR: Curtain gas
DIO: Diet-induced obesity
FC: Fold change
FDR: False discovery rate
FeNO: Fractional exhaled nitric oxide
FEV_1_: Forced expiratory volume in 1 s
FEV_1_/FVC: Ratio of FEV_1_ to FVC
FVC: Forced vital capacity
FMO: Fluorescence minus one
GBD: Global Burden of Disease
GINA: Global Initiative for Asthma
GP: Glycerophospholipid
HDM: House dust mite
H&E: Hematoxylin and eosin
HFD: High-fat diet
H-NWA: Normal-weight asthma patients (human)
H-OA: Overweight/obese asthma patients (human)
i.n.: intranasal
i.p.: intraperitoneal
IQR: Interquartile range
KEGG: Kyoto Encyclopedia of Genes and Genomes
LPA: Lysophosphatidic acid
LPC: Lysophosphatidylcholine
LPE: Lysophosphatidylethanolamine
LPG: Lysophosphatidylglycerol
LPI: Lysophosphatidylinositol
MFI: Mean fluorescence intensity
M-NWA: Normal-weight asthma mice
M-OA: Obese asthma mice
MRM: Multiple reaction monitoring
MTBE: Methyl tert-butyl ether
N/A: Not available
NCD: Normal chow diet
PAS: Periodic acid-Schiff
PA: Phosphatidic acid
PC: Phosphatidylcholine
PE: Phosphatidylethanolamine
PG: Phosphatidylglycerol
PI: Phosphatidylinositol
PCA: Principal component analysis
PLS-DA: Partial least squares discriminant analysis
ROI: Region of interest
RV: Residual volume
RV/TLC: Ratio of residual volume to total lung capacity
SD: Standard deviation
SPF: Specific pathogen-free
TCR: T cell receptor
Th: T helper
TLC: Total lung capacity
UPLC–MS/MS: Ultra-Performance Liquid Chromatography–Tandem Mass Spectrometry
VIP: Variable Importance in Projection
