# Supplementary material for: Lysophosphatidylcholine 18:2 exacerbates Th17-dominant inflammation in obese asthma
Source: Lipids Health Dis. 2026 Feb 24;25:95. doi: 10.1186/s12944-026-02907-4 (PMC13037044; doi:10.1186/s12944-026-02907-4)
Supplement: Supplementary file 3 — Additional file 3: Supplementary Methods. This file provides comprehensive and step-by-step protocols for the additional experimental methodologies referenced in the manuscript. [file 12944_2026_2907_MOESM3_ESM.docx]

**Supplemental methods**

***ELISA***

IL-17A concentrations were quantified using a commercial ELISA kit (Elabscience, E-EL-M0047-48) following the manufacturer’s protocol. Briefly, standards and cell culture supernatants were loaded into wells pre-coated with capture antibodies and incubated at 37 °C for 2 h. Afterward, wells were incubated sequentially with a biotinylated detection antibody, streptavidin–HRP conjugate, and TMB substrate. The reaction was stopped with stop solution, and absorbance was recorded at 450 nm within 15 minutes.

***Western blotting***

For Western blotting, whole-cell lysates were extracted using RIPA lysis buffer (Beyotime, P0013B) supplemented with protease and phosphatase inhibitors (Epizyme, GRF103). After incubation on ice for 30 minutes, samples were centrifuged at 12,000 rpm for 10 minutes at 4 °C. The supernatants were then collected, mixed with 5× loading buffer, and heated at 100 °C for 10 minutes. Proteins were resolved by SDS-PAGE and transferred onto PVDF membranes. Membranes were blocked and incubated overnight at 4 °C with primary antibodies against ROR**γ**t (1:1000, Proteintech, 29910-1-AP) and β-actin (1:5000, Proteintech, 20536-1-AP). After washing, HRP-conjugated goat anti-rabbit IgG secondary antibody (1:5000, CST, 7074P2) was applied, and protein bands were visualized using a chemiluminescent detection system.

***Real-time quantitative PCR (RT–qPCR)***

RNA was extracted from iTh17 cells using TRIzol reagent (Thermo Fisher Scientific, Waltham, USA), and reverse-transcribed into cDNA. Quantitative PCR was conducted on a QuantStudio 5 Real-Time PCR System (Thermo Fisher Scientific) using TB Green Premix Ex Taq™ II (TaKaRa, RR820A). Expression levels of target genes were normalized to *Actb* and quantified using the comparative Ct (2^⁻ΔΔCt^) method.

Sequences of real-time PCR primers (5’ to 3’):

| Gene | Forward | Reverse |
| --- | --- | --- |
| *Il17a* | TCAGCGTGTCCAAACACTGAG | CGCCAAGGGAGTTAAAGACTT |
| *Il17f* | TGCTACTGTTGATGTTGGGAC | CAGAAATGCCCTGGTTTTGGT |
| *Rorc* | GACCCACACCTCACAAATTGA | AGTAGGCCACATTACACTGCT |
| *Pparg* | CTCCAAGAATACCAAAGTGCGA | GCCTGATGCTTTATCCCCACA |
| *Cd36* | CAAAACCCAGATGACGTGGC | TCCTCGGGGTCCTGAGTTAT |
| *Lpl* | TTGCCCTAAGGACCCCTGAA | TTGAAGTGGCAGTTAGACACAG |
| *Plin5* | CTTCCTGCCCATGACTGAGG | GACCCCAGACGCACAAAGTAG |
| *Fabp4* | AAGGTGAAGAGCATCATAACCCT | TCACGCCTTTCATAACACATTCC |
| *Scd1* | TTCCCTCCTGCAAGCTCTAC | CAGAGCGCTGGTCATGTAGT |
| *Scd2* | GATCTCTGGCGCTTACTCAGC | CTCCCCAGTGGTGAGAACTC |
| *Actb* | GTGACGTTGACATCCGTAAAGA | GCCGGACTCATCGTACTCC |

***CCK-8 cell viability assay***

Cell viability was assessed using the CCK-8 assay (LABLEAD, CK001). iTh17 cells were plated into 96-well plates at a density of 5 × 10^4^ cells per well and incubated with varying concentrations of LPC 18:2 (0, 10, 20, 50, 100, and 200 μM) for 72 hours. After treatment, 10 μL of CCK-8 reagent was added to each well (final volume 110 μL). The plates were then incubated at 37 °C for 2 hours to allow formazan dye development. Absorbance was measured at 450 nm using a microplate reader to determine cell viability.

***Apoptosis assay***

Apoptosis of iTh17 cells was assessed using the Annexin V-FITC/PI Apoptosis Detection Kit (Keygen Bio, KGA106) following the manufacturer’s instructions. Cells were stained with Annexin V-FITC and propidium iodide (PI), and apoptosis was analyzed by flow cytometry.

***Autotaxin (ENPP2) concentration by ELISA***

Autotaxin protein levels were quantified using commercial sandwich ELISA kits. For human samples, plasma autotaxin concentrations were measured using a human ENPP2/autotaxin ELISA kit (Elabscience, E-EL-H6296) according to the manufacturer’s instructions. Venous blood was collected from asthma patients. Plasma was isolated by centrifugation, and aliquots were stored at −80 °C until analysis. Plasma samples from normal-weight (H-NWA) and overweight/obese (H-OA) asthma patients were thawed on ice, diluted 1:40 in sample diluent as recommended by the manufacturer, and processed on the same ELISA plate. Autotaxin concentrations were calculated using a standard curve generated from serial dilutions of the standards and reported in ng/mL.

In the murine HDM-induced asthma model, autotaxin was measured in lung homogenates and bronchoalveolar lavage fluid (BALF). For lung homogenates, snap-frozen lung tissue was homogenized in ice-cold lysis buffer, clarified by centrifugation, and the supernatant was collected for analysis. Total protein concentrations in lung homogenate supernatants were determined using a BCA protein assay kit (Epizyme, ZJ102), and autotaxin levels were measured with a mouse autotaxin ELISA kit (Abmart, AB-K332302A). Results were reported as ng/mg total protein. For BALF collection, mice were deeply anesthetized, a tracheal cannula was inserted, and the lungs were gently lavaged three aliquots of 0.8 mL sterile PBS. The recovered fractions were pooled, centrifuged to remove cells, and the supernatants were stored at −80 °C until analysis. Autotaxin concentrations in BALF were determined with the same Abmart ELISA kit (AB-K332302A) and reported in ng/mL. Values below the lower limit of detection were recorded as undetectable.

***Autotaxin (ENPP2) lysoPLD activity assay***

Autotaxin (ENPP2) lysoPLD activity in plasma from asthma patients was measured using a fluorogenic kinetic assay kit (Echelon Biosciences, K-4100) according to the manufacturer’s instructions. Plasma aliquots were thawed on ice, clarified by brief centrifugation to remove particulates, and equilibrated at room temperature before analysis. Samples were assayed in duplicate in black 96-well plates. Reaction mixtures containing the fluorogenic substrate FS-3 and lysophosphatidylcholine were prepared immediately before use and protected from light. Wells were mixed gently on an orbital shaker, avoiding bubble formation, and fluorescence was recorded kinetically at 37 °C (excitation 485 nm, emission 528 nm) every 2 min for 2 h using a fluorescence microplate reader. Because plasma components can affect fluorescence, fluorescein control wells prepared in the corresponding sample matrix were run in parallel to derive a sample-specific correction factor. Reaction rates (RFU/min) were calculated by linear regression from the linear portion of the kinetic trace (20–100 min), and duplicate slopes were averaged. Slopes were converted to autotaxin activity units using the fluorescein calibration and corrected by subtracting spontaneous FS-3 hydrolysis measured in parallel. Autotaxin activity is reported as autotaxin units, with one unit defined as pM FS-3 hydrolyzed per minute under these assay conditions.
