# Supplementary material for: Lysophosphatidylcholine 18:2 exacerbates Th17-dominant inflammation in obese asthma
Source: Lipids Health Dis. 2026 Feb 24;25:95. doi: 10.1186/s12944-026-02907-4 (PMC13037044; doi:10.1186/s12944-026-02907-4)
Supplement: Supplementary file 4 — Additional file 4: Supplementary Figures S1–S6 and Supplementary Table S1. Figure S1. presents FMO and isotype controls used for flow cytometry gating of CD4⁺ T cell subsets. Figure S2. shows serum LPC 18:2 levels stratified by pre-bronchodilator FEV₁% predicted and FEV₁/FVC in asthma patients. Figure S3. documents the flow cytometric validation of naïve CD4⁺ T cell isolation from mouse spleens. Figure S4. evaluates the effects of LPC 18:2 on cell viability and apoptosis in iTh17 cells. Figure S5. summarizes autotaxin and LPA profiles in obese versus non-obese asthma in human and murine samples. Figure S6. illustrates the impact of pharmacological inhibition of the autotaxin–LPA axis on LPC 18:2–induced Th17 differentiation in vitro. Supplementary Table S1 presents correlation analyses of LPC 18:2 levels with asthma-related parameters. [file 12944_2026_2907_MOESM4_ESM.docx]

**Supplementary figures and table**

**
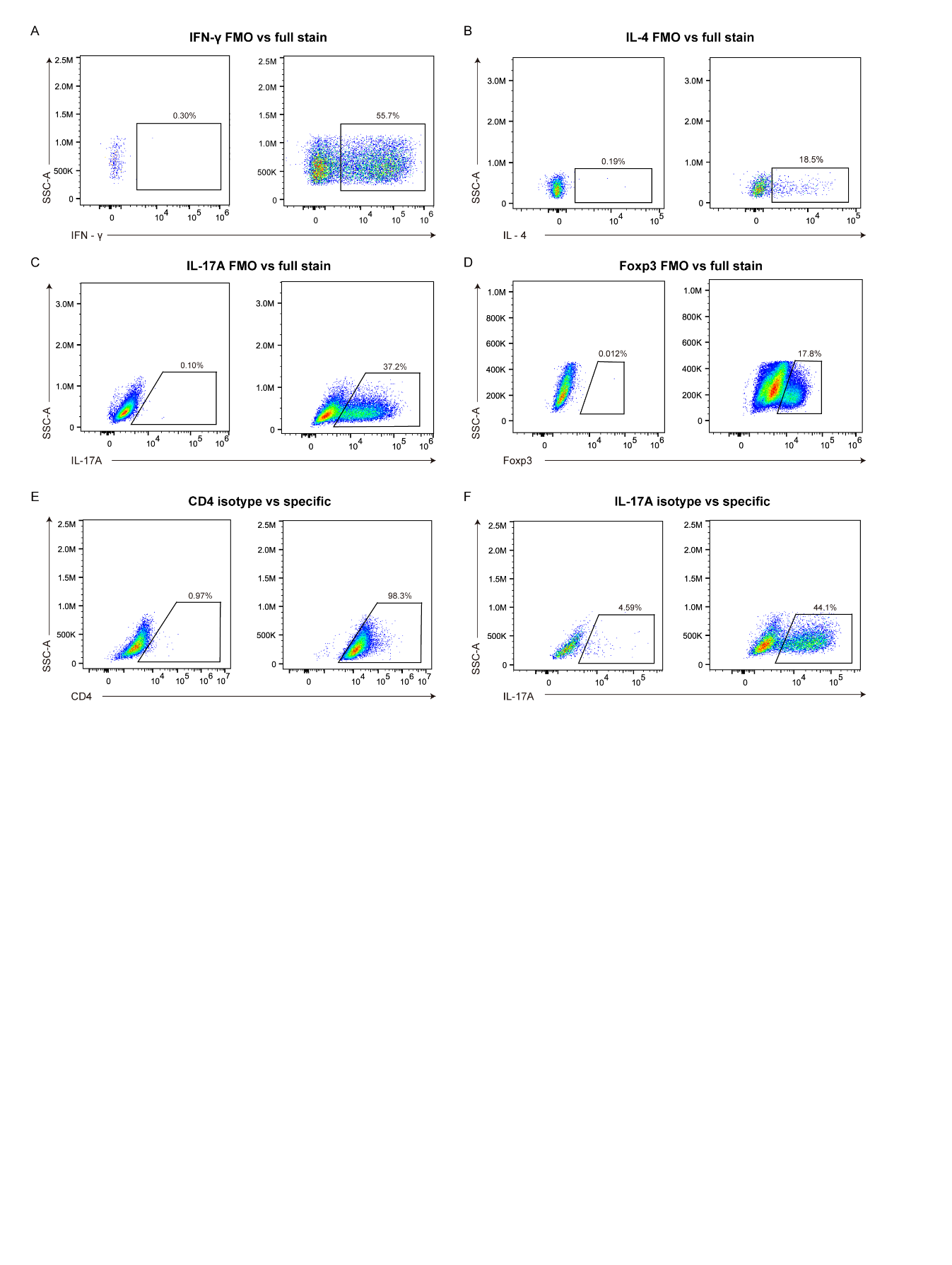
**

Supplementary Figure S1. Representative FMO and isotype controls used for flow cytometry gating of CD4⁺ T-cell subsets. A–D Representative FMO controls for IFN-γ, IL-4, IL-17A, and Foxp3, with the corresponding full-stain samples, illustrating how positive gates were defined. Each FMO control contained the full antibody panel except for the marker of interest, and gates were set based on the corresponding FMO distributions to distinguish positive events from background. E–F Representative isotype-matched controls for anti-CD4 and anti-IL-17A, compared with specific antibody staining, demonstrating minimal non-specific binding.


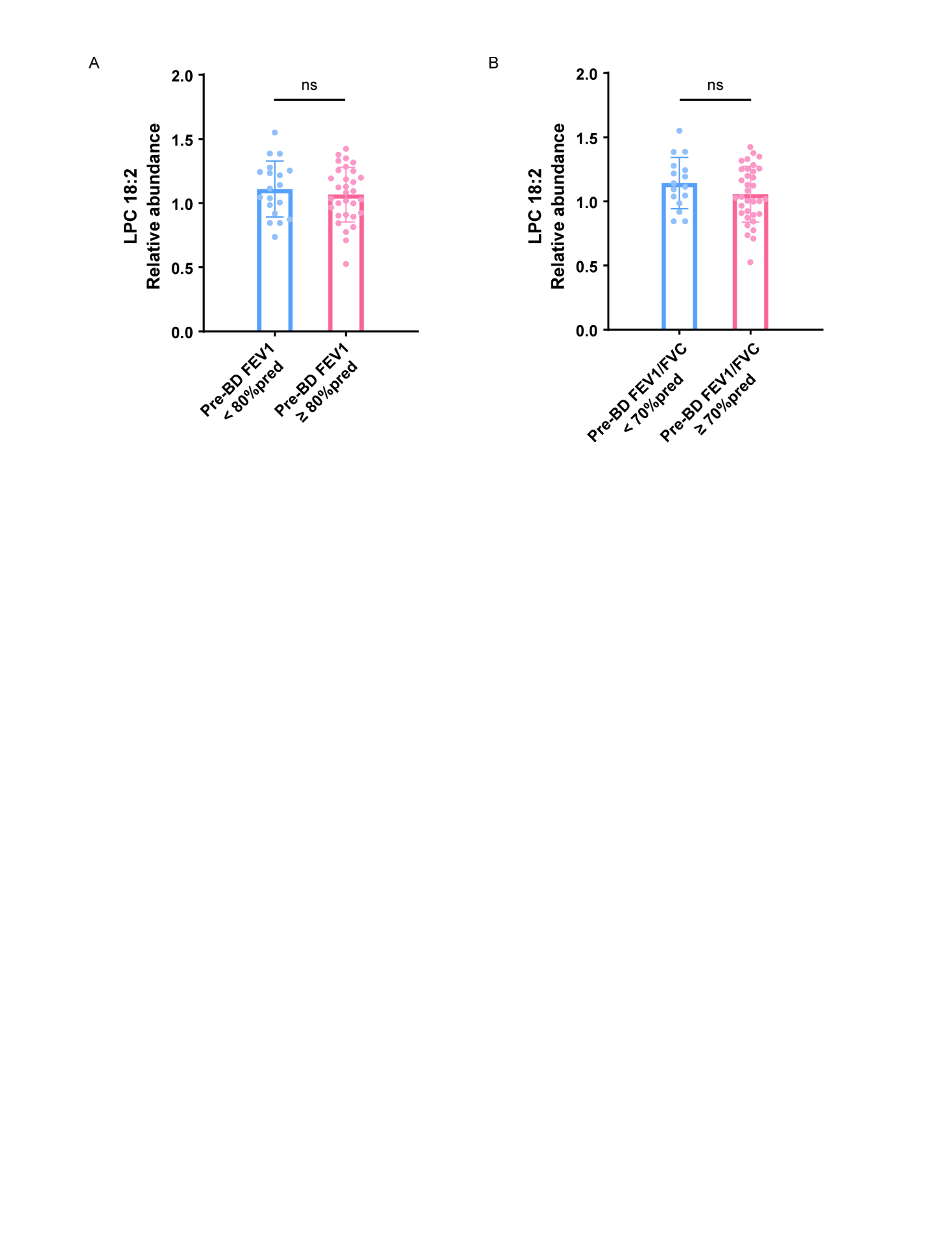

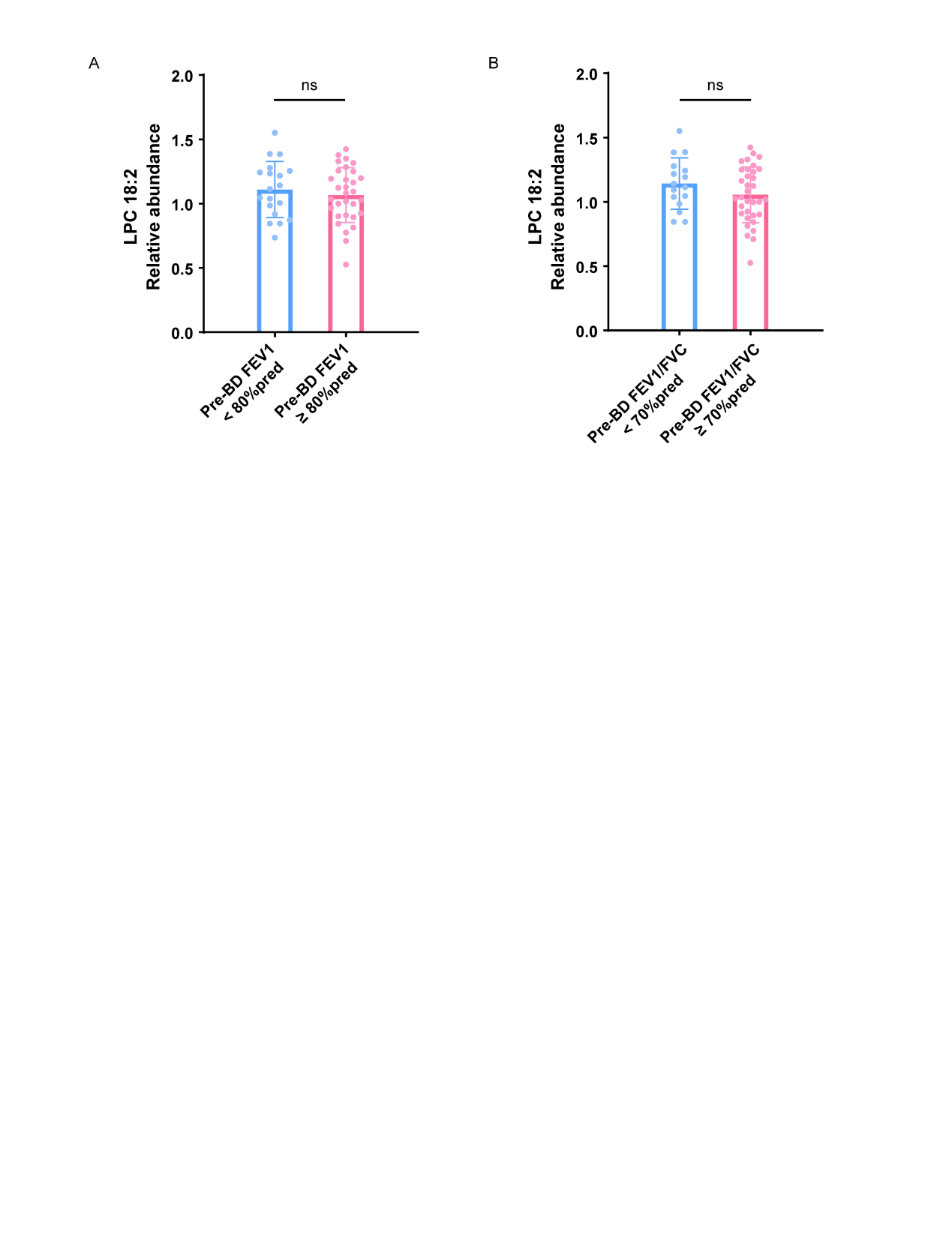


Supplementary Figure S2. Serum LPC 18:2 levels by lung function in asthma patients. A Serum LPC 18:2 levels stratified by pre-bronchodilator (pre-BD) FEV₁% predicted (≥ 80% vs. < 80%). B Serum LPC 18:2 levels stratified by pre-bronchodilator FEV₁/FVC (≥ 70% vs. < 70%). Data are presented as mean ± SD. Statistical differences were assessed using unpaired two-tailed *t*-tests; both comparisons were not significant (*P* > 0.05). ns, not significant.


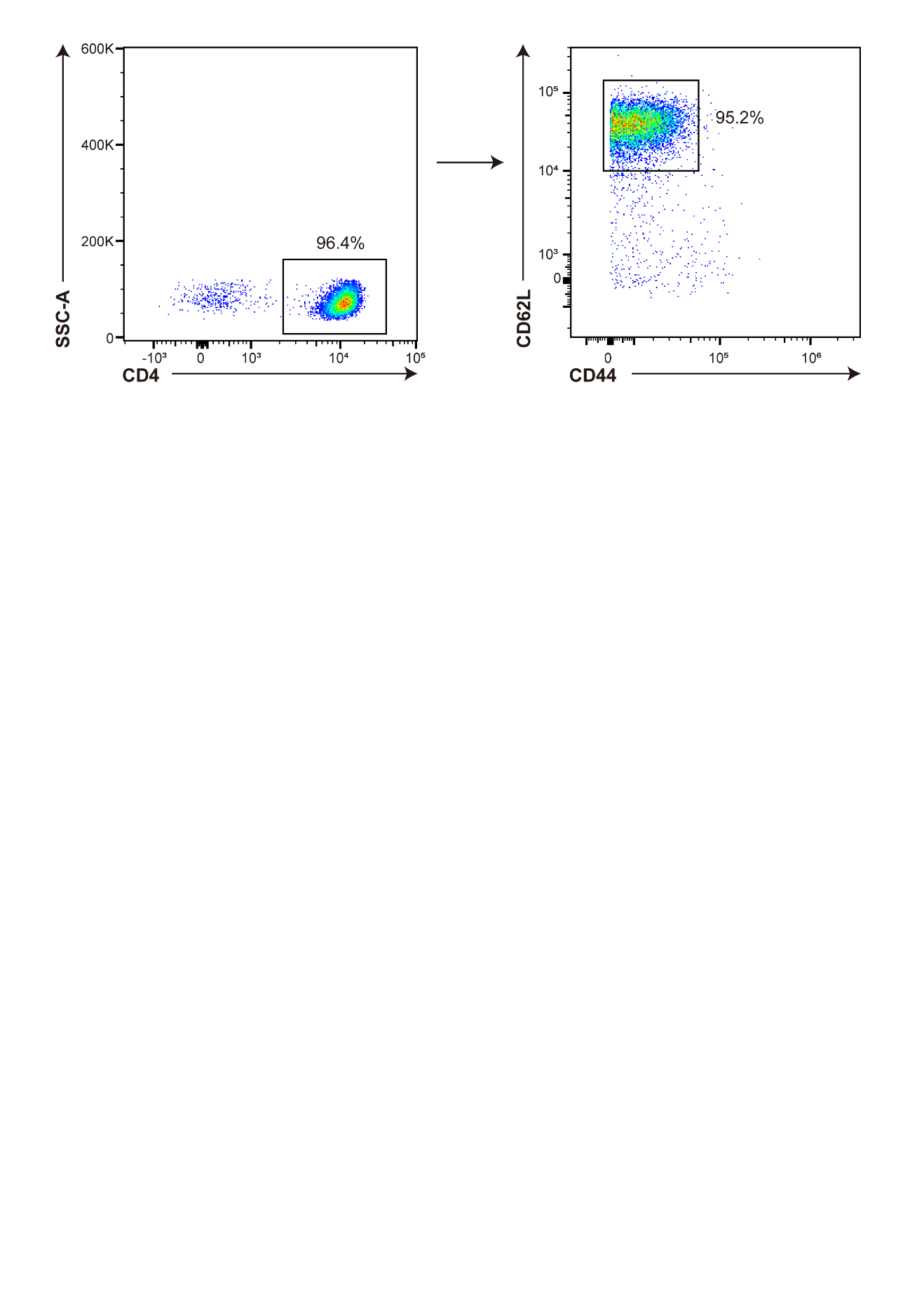


Supplementary Figure S3. Flow cytometric assessment of naïve CD4⁺ T cell isolation purity. Naïve CD4⁺ T cells were isolated from mouse spleens using magnetic bead sorting and identified as CD4⁺CD62L⁺CD44⁻ by flow cytometry. The purity of isolated naïve CD4⁺ T cells exceeded 90% in all samples.


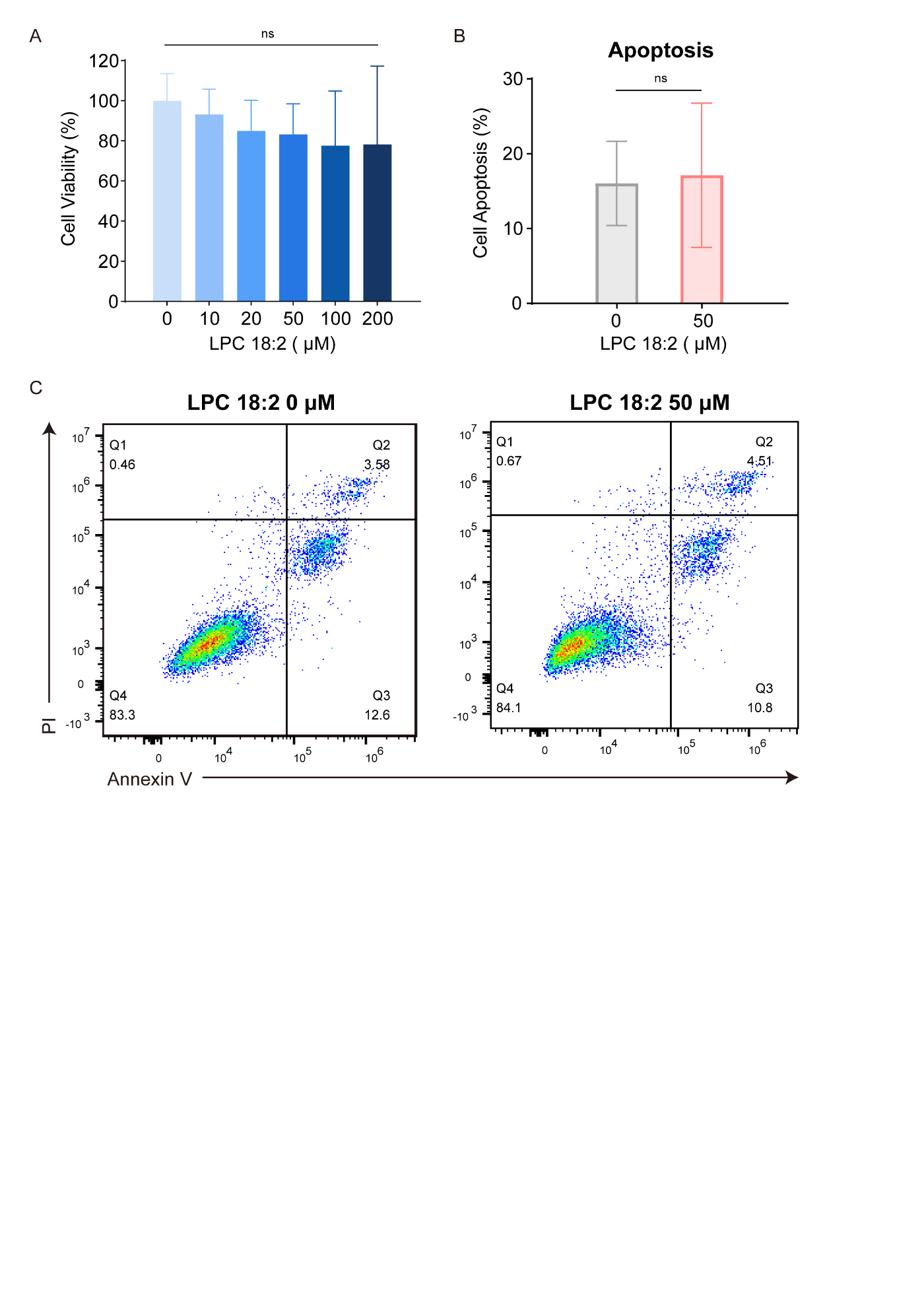


Supplementary Figure S4. Effects of LPC 18:2 on cell viability and apoptosis in iTh17 cells. A Cell viability was assessed by the CCK-8 assay after treatment with LPC 18:2 at 0, 10, 20, 50, 100, or 200 μM. B–C Flow cytometric analysis of apoptosis in iTh17 cells treated with 0 or 50 μM LPC 18:2. Data are presented as mean ± SD. ns, not significant.


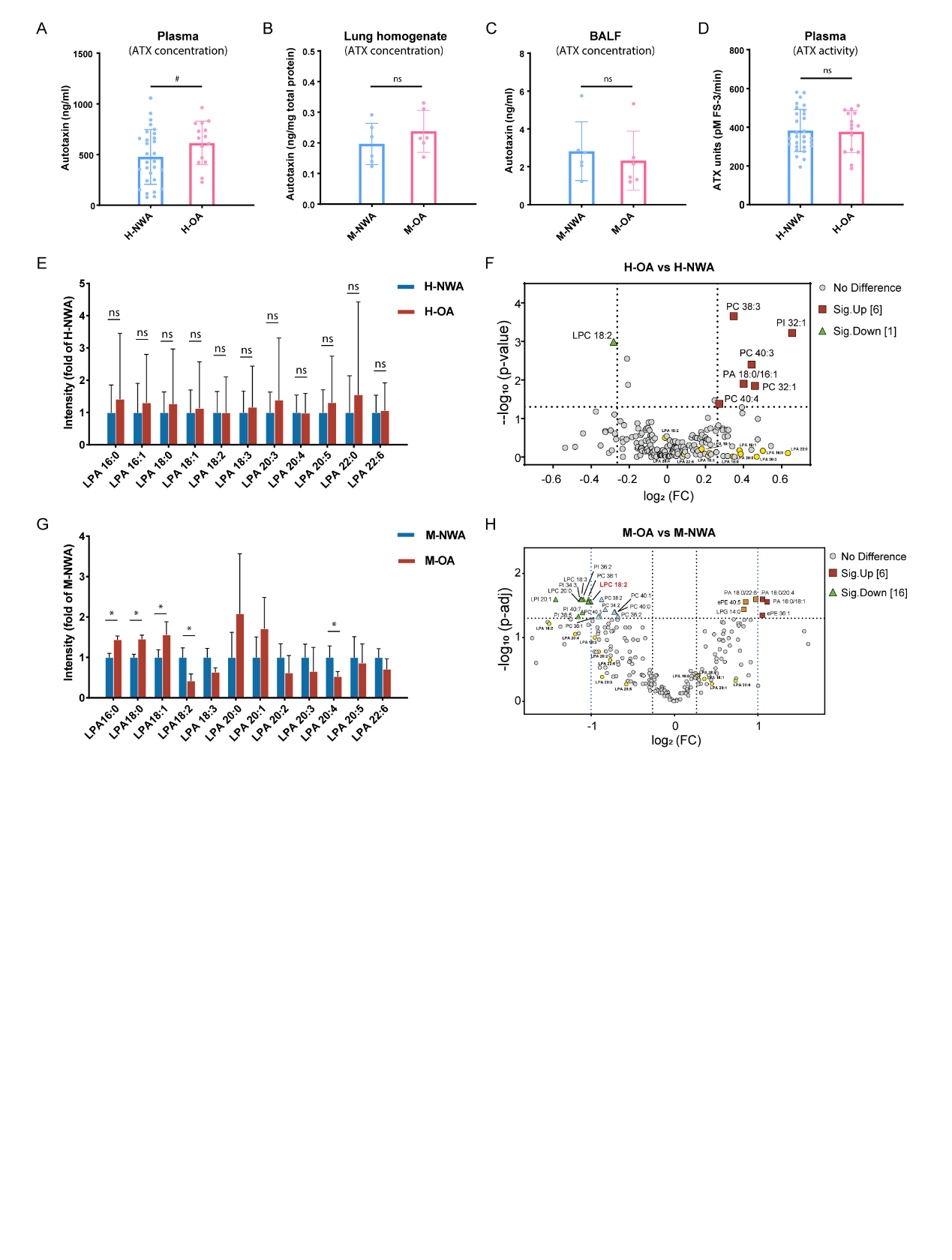

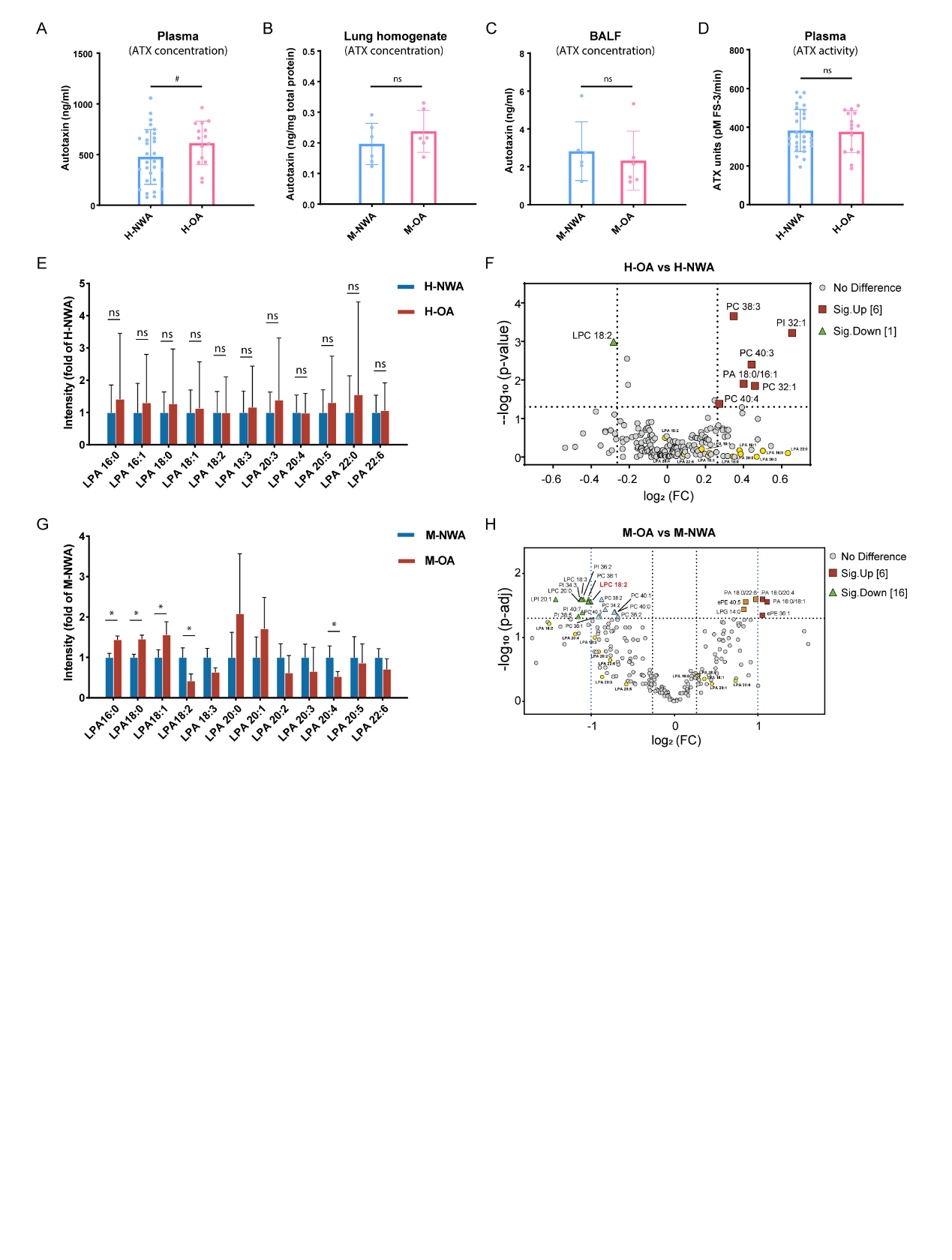


Supplementary Figure S5. Autotaxin and LPA profiles in overweight/obese versus normal-weight asthma. A Plasma autotaxin (ATX/ENPP2) concentrations in normal-weight (H-NWA, n = 29) and overweight/obese (H-OA, n = 15) asthma patients. B Autotaxin levels in lung homogenates from normal-weight (M-NWA, n = 6) and obese (M-OA, n = 6) asthmatic mice, expressed as ng/mg total protein. C Autotaxin concentrations in bronchoalveolar lavage fluid (BALF) from M-NWA (n = 6) and M-OA mice (n = 6). D Plasma autotaxin lysoPLD activity measured by a fluorogenic kinetic assay in normal-weight (H-NWA, n = 29) and overweight/obese (H-OA, n = 15) asthma patients. E Relative abundances of individual serum LPA species in H-NWA and H-OA patients, expressed as fold change relative to the mean of the H-NWA group. F Volcano plot of the human discovery lipidomic dataset; dashed lines indicate the exploratory thresholds for differential abundance (|fold change| > 1.2 and nominal *P* < 0.05). Annotated LPA species are highlighted in yellow. G Relative abundances of serum LPA species in M-NWA and M-OA mice, expressed as fold change relative to the mean of the M-NWA group. H Volcano plot of the murine confirmatory lipidomic dataset; dashed lines indicate the stringent criteria used for significance (|fold change| > 2.0 and FDR-adjusted *P* < 0.05). Annotated LPA species are highlighted in yellow. Data are presented as mean ± SD. Statistical differences were assessed using an unpaired two-tailed *t*-test for panel A and D and the Mann–Whitney *U* test for panels B, C, E, and G. **P* < 0.05, #*P* = 0.05–0.10, ns, not significant.


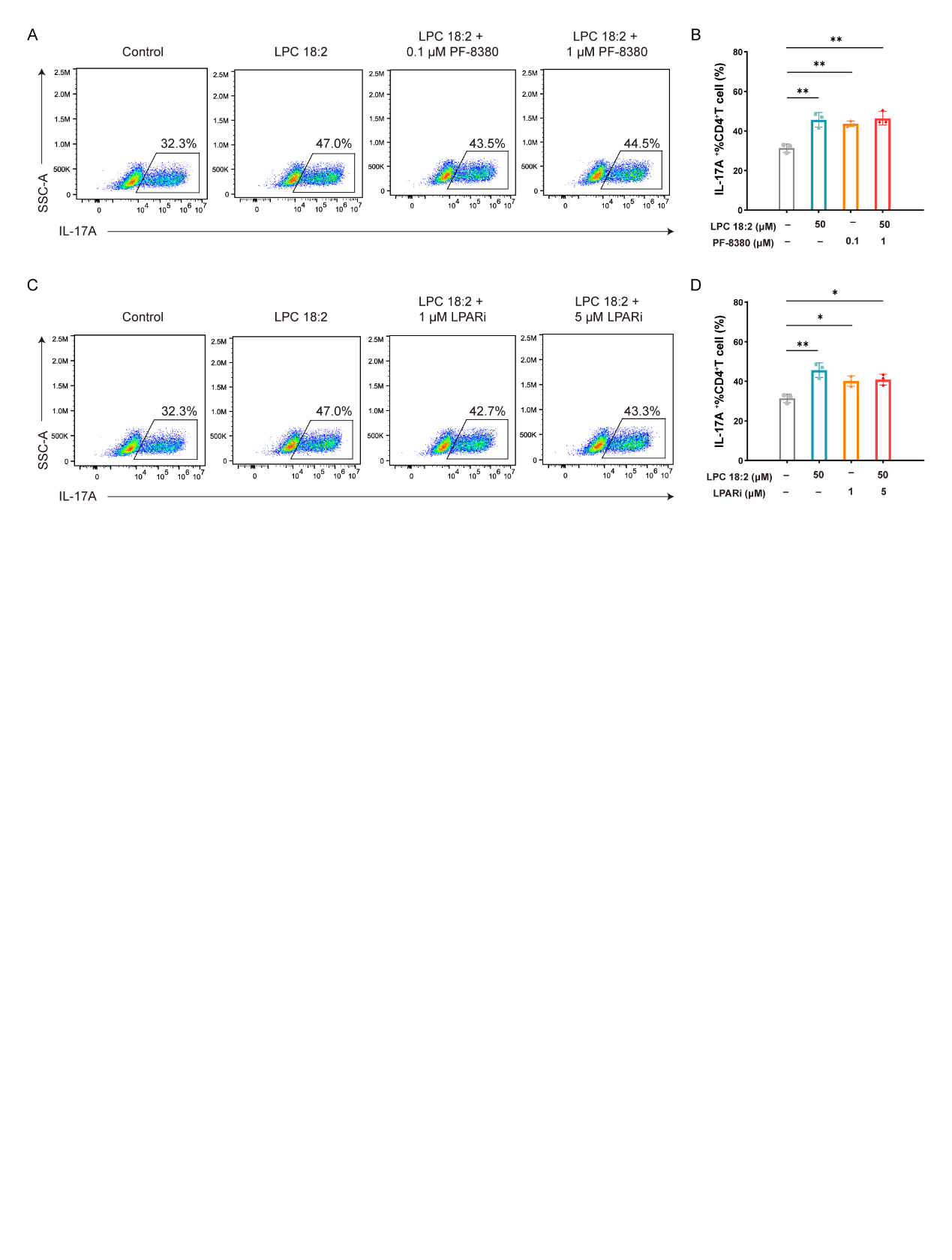

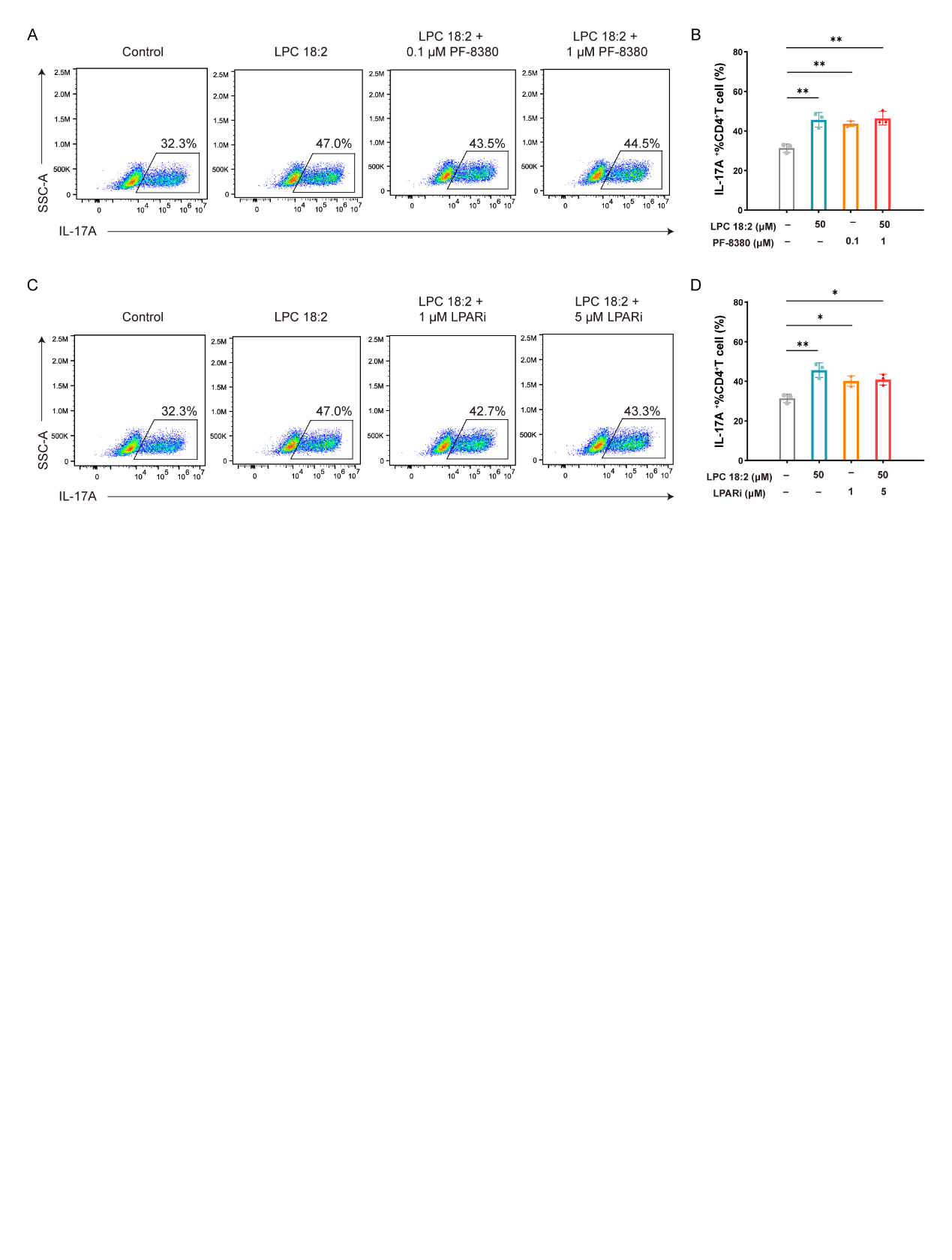


Supplementary Figure S6. Pharmacological inhibition of the autotaxin–LPA axis does not attenuate the Th17-promoting effect of LPC 18:2. A Representative flow cytometry plots of IL-17A expression in CD4⁺ T cells differentiated under iTh17-polarizing conditions. Cells were treated with LPC 18:2 (50 μM) alone or in combination with PF-8380 (0.1 or 1 μM), with 0 μM LPC 18:2 (vehicle) as the control. B Quantification of IL-17A⁺CD4⁺ T-cell frequencies under the conditions shown in (A). C Representative flow cytometry plots of IL-17A expression in CD4⁺ T cells differentiated under iTh17-polarizing conditions. Cells were treated with LPC 18:2 (50 μM) alone or in combination with LPA receptor antagonists (LPARi; Ki16425 and H2L5186303; 1 or 5 µM each), with 0 μM LPC 18:2 (vehicle) as the control. D Quantification of IL-17A⁺CD4⁺ T-cell frequencies under the conditions shown in (C). Data are presented as mean ± SD. Statistical analyses were performed using one-way ANOVA followed by Tukey’s post hoc test. **P* < 0.05, ***P* < 0.01, ****P* < 0.001.

Table S1. Correlations between serum LPC 18:2 levels and clinical parameters in asthma patients.

| Parameter | *ρ* | *P*-value |
| --- | --- | --- |
| ACT score | 0.146 | 0.311 |
| Blood eosinophils (/µL) | 0.077 | 0.595 |
| Total IgE (IU/mL) | 0.182 | 0.221 |
| FeNO (ppb) | 0.224 | 0.135 |
| FEV_1_% predicted | -0.078 | 0.589 |
| FEV₁/FVC ratio | -0.110 | 0.448 |

Note: Data are presented as Spearman’s rank correlation coefficients (*ρ*) and *P* values for associations between serum LPC 18:2 levels and clinical parameters among asthma patients. None of the correlations reached statistical significance (all *P* > 0.05).
