# Supplementary material for: Lysophosphatidylcholine 18:2 exacerbates Th17-dominant inflammation in obese asthma
Source: Lipids Health Dis. 2026 Feb 24;25:95. doi: 10.1186/s12944-026-02907-4 (PMC13037044; doi:10.1186/s12944-026-02907-4)
Supplement: Supplementary file 5 — Additional file 5: Supplementary Figure S7. This file contains the original, uncropped Western blot membranes for RORγt and β-actin corresponding to Fig. 5D. [file 12944_2026_2907_MOESM5_ESM.docx]

**Original Images for Blot**


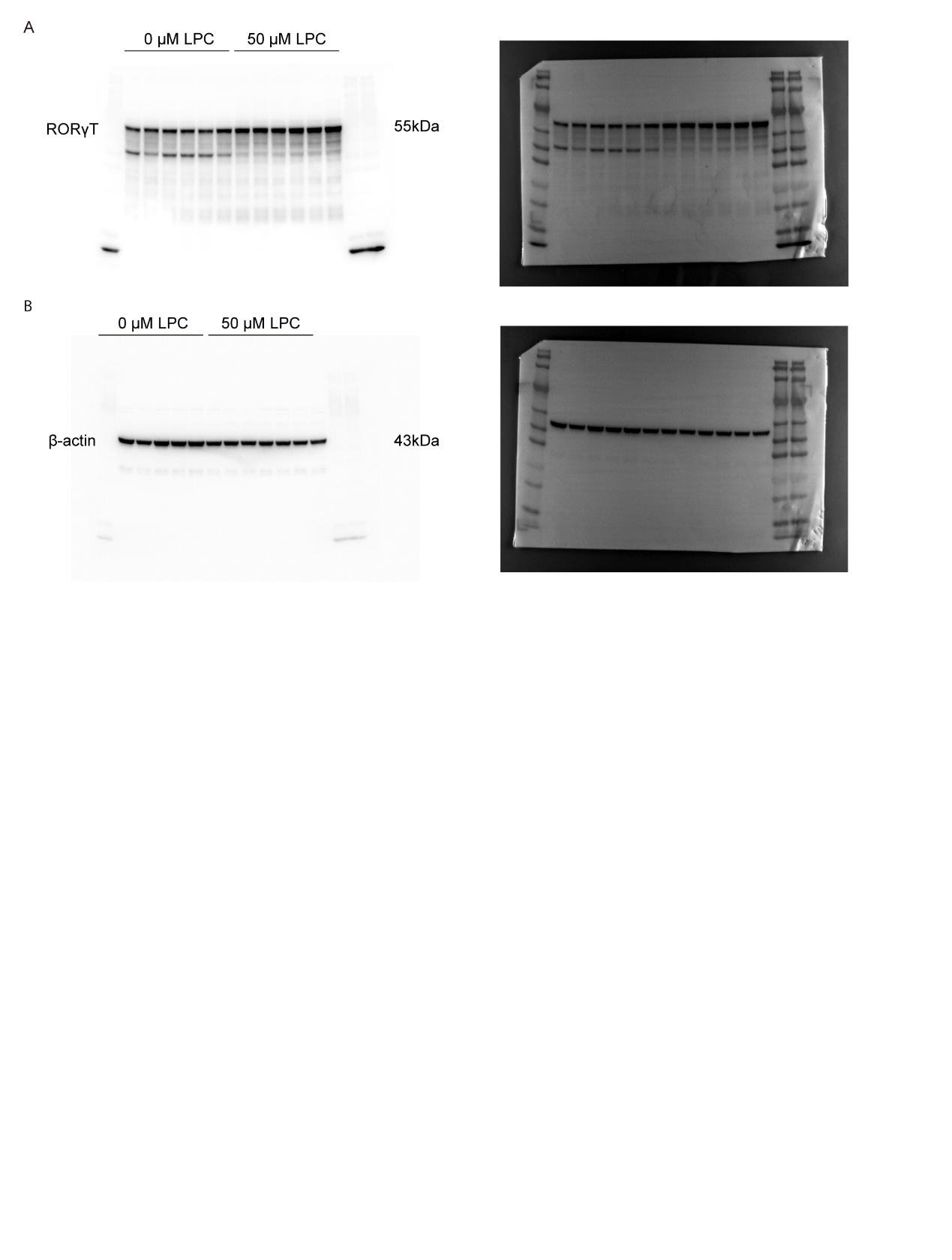


Supplementary Figure S7. Western blot analysis of RORγt protein expression in iTh17 cells treated with 0 or 50 µM LPC 18:2. A Representative Western blot showing RORγt. B The same membrane shown in (A) was stripped and re-probed for β-actin as a loading control. The full, uncropped membrane presented here corresponds to the cropped version shown in Fig. 5D in the main text.
